# Seaweed and Seaweed-Based Functional Metabolites as Potential Modulators of Growth, Immune and Antioxidant Responses, and Gut Microbiota in Fish

**DOI:** 10.3390/antiox12122066

**Published:** 2023-12-01

**Authors:** Muhammad A. B. Siddik, Prue Francis, Md Fazle Rohani, Mohammed Shariful Azam, Thomas S. Mock, David S. Francis

**Affiliations:** 1 School of Life and Environmental Sciences, Deakin University, Geelong, VIC 3216, Australia; prue.francis@deakin.edu.au (P.F.); tom.mock@deakin.edu.au (T.S.M.); d.francis@deakin.edu.au (D.S.F.); 2 Department of Aquaculture, Bangladesh Agricultural University, Mymensingh 2202, Bangladesh; rohani_aq@bau.edu.bd; 3 Department of Fisheries (DoF), Ramna, Dhaka 1205, Bangladesh; sharif@fisheries.gov.bd

**Keywords:** bioactive compounds, gut microbiota, polyphenols, polysaccharides, pigments, omega-3 fatty acids, sustainable aquaculture

## Abstract

Seaweed, also known as macroalgae, represents a vast resource that can be categorized into three taxonomic groups: Rhodophyta (red), Chlorophyta (green), and Phaeophyceae (brown). They are a good source of essential nutrients such as proteins, minerals, vitamins, and omega-3 fatty acids. Seaweed also contains a wide range of functional metabolites, including polyphenols, polysaccharides, and pigments. This study comprehensively discusses seaweed and seaweed-derived metabolites and their potential as a functional feed ingredient in aquafeed for aquaculture production. Past research has discussed the nutritional role of seaweed in promoting the growth performance of fish, but their effects on immune response and gut health in fish have received considerably less attention in the published literature. Existing research, however, has demonstrated that dietary seaweed and seaweed-based metabolite supplementation positively impact the antioxidant status, disease resistance, and stress response in fish. Additionally, seaweed supplementation can promote the growth of beneficial bacteria and inhibit the proliferation of harmful bacteria, thereby improving gut health and nutrient absorption in fish. Nevertheless, an important balance remains between dietary seaweed inclusion level and the resultant metabolic alteration in fish. This review highlights the current state of knowledge and the associated importance of continued research endeavors regarding seaweed and seaweed-based functional metabolites as potential modulators of growth, immune and antioxidant response, and gut microbiota composition in fish.

## 1. Introduction

Aquaculture has become an essential source of seafood for the human population and contributes significantly to global food security. With the demand for nutritious seafood increasing due to population growth and declining wild fish stocks, aquaculture has helped bridge the gap between supply and demand [1]. However, continually increasing aquafeed costs and disease outbreaks have become common challenges impacting the profitability and sustainability of this sector [2,3]. As such, aquaculture researchers globally are looking for more affordable and health-promoting feed ingredients that can sustain fish growth as well as prevent disease in aquaculture production. In recent years, there has been a growing interest in utilizing various seaweeds, including red seaweed (e.g., *Gracilaria*, *Porphyra*), brown seaweed (e.g., *Laminaria*, *Ascophyllum*), and green seaweed (e.g., *Ulva*, formerly *Enteromorpha*), as potential sources of bioactive compounds and feed ingredients for inclusion in aquafeed given their favorable nutritional composition, environmental sustainability, and potential health-promoting factors for farmed fish [4]. A growing number of studies have indicated that seaweed and seaweed-based functional metabolites improve serum immune and antioxidant status [5] and disease resistance [6] in fish when incorporated in aquafeed as a supplement.

Seaweed is a valuable source of protein, ranging from 11% to 32% in its composition (dry weight, DW), with an excellent combination of essential amino acids, soluble dietary fiber, minerals, and vitamins suitable for use in aquafeed formulations [4,7,8]. However, the protein and its essential amino acid levels have been shown to be low and variable depending on species, growing conditions, season of harvest, water depth, salinity of water, processing methods, and geographical conditions [9]. Previous research has shown that low inclusion levels (~5%) of seaweed or their bioactive metabolites in fish diets evoke positive effects on growth performance [10], while inclusion levels (~10%) in fish diets neither improve nor decrease growth performance in fish [11]. Similarly, Soler-vila et al. [12] reported that up to 10% inclusion of red alga (*Porphyra dioica*) in a practical diet does not compromise growth, whilst an inclusion level of 15% reduced growth performance in rainbow trout (*Oncorhynchus mykiss*). However, the nutrient utilization efficiency of seaweed in aquafeed is dependent on the feeding habits of cultured species and the inclusion rate [13]. For instance, in carnivorous species like European seabass (*Dicentrarchus labrax*), the optimum inclusion level of *Ulva lactuca* has been reported as being 5% of the diet [14], while in omnivorous species like Nile tilapia (*Oreochromis niloticus*), this seaweed species can constitute up to 20% of practical diets with no detrimental effects on growth performance [15]. Nevertheless, seaweed is also a good source of omega-3 fatty acids, particularly eicosapentaenoic acid (EPA) and docosahexaenoic acid (DHA), in their fatty acid profile [16] in comparison with terrestrial plants. These fatty acids are essential for the metabolic activity of fish as well as maintaining the structural integrity and the fluidity and permeability of cellular membranes [17].

Recent research findings have revived interest in seaweed as a safe alternative to preventive and therapeutic drugs in farmed fish to reduce economic loss caused by infectious diseases [6]. Seaweed has a wide range of bioactive substances, including polyphenols, pigments, essential fatty acids, vitamins, and amino acids [18], where seaweed polyphenols are reported to enhance the immune response and disease resistance in fish [19,20]. Thanigaivel et al. [21] reported that dietary administration of total phenolic compounds (TPC) from *Gracilaria foliifera* (Rhodophyta) and *Sargassum longifolium* (Phaeophyceae) at inclusion levels ranging from 14. 71 mg/g GAE to 18.42 mg/g GAE (gallic acid equivalent) in Mozambique tilapia (*Oreochromis mossambicus*) increased percent survival against *Aeromonas salmonicida* infection. However, the existing literature mostly deals with seaweed and seaweed extract as aquafeed supplements for fish. There are currently very few studies on how seaweed-derived bioactive compounds can supplement aquafeed with the goal of improving fish health. Enhanced lysozyme and immune-related gene expression and improved stress response and disease resistance have also been reported in various fish fed seaweed-supplemented diets [6,22]. However, the immunostimulatory properties of seaweed also depend on the level of inclusion in the diet. For example, Vazirzadeh et al. [22] reported a significant increase in lysozyme activity in rainbow trout when supplemented with 5% *Gracilariopsis persica* (Rhodophyta) in the diet, while 10% inclusion had no effect following an 83-day feeding trial. These findings suggest that seaweed provides health benefits when fed to fish at optimized levels and, therefore, has potential as a functional ingredient in fish feeds. Furthermore, seaweed may enhance fish gut health by altering gut microbiota, thus strengthening the gut barrier function. For instance, an abundance of beneficial bacteria such as *Shewanella* sp. has been reported when adding seaweed (*Asparagopsis taxiformis* (Rhodophyta) extract to feeds for Atlantic salmon (*Salmo salar*) [23]. Also, augmented villus height and increased intestinal goblet cell numbers have been reported following algae (*Spirulina platensis*) supplementation in fish [24].

Despite many positive attributes, seaweed has seldom been researched as a source of functional metabolites in aquafeed for fish. The potential effects of dietary seaweed and the inclusion of seaweed-derived bioactive compounds on intestinal micromorphology, gut microbiota composition, and disease resistance in fish have, to date, received scant attention in the published literature. Further, the variation in the nutritional composition of various seaweed, the availability of seaweed, and the digestibility and palatability issues of seaweed at higher inclusion levels have not been significantly focused on in previous research. Therefore, this review compares and illustrates the nutritional compositions of various seaweeds and the potential impacts of seaweed and seaweed-based secondary metabolites on growth performance, feed utilization, gut microbiota composition, immunity, and disease resistance in fish. The present review also highlights the current barriers that are hindering seaweeds from being exploited as a feed component in aquafeeds and the possible solutions to overcome these obstacles.

## 2. Seaweed Resources and Seaweed’s Nutritional Composition

**Figure 1:**
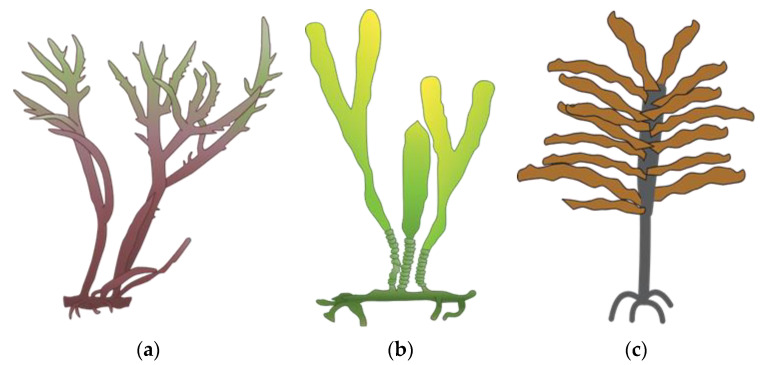
Schematic representation of (**a**) red (Rhodophyta), (**b**) green (Chlorophyta), and (**c**) brown (Phaeophyceae) seaweed utilized as feed additives and sources of bioactive compounds in aquafeed for fish. Seaweed drawings are courtesy of the Integration and Application Network, University of Maryland Center for Environmental Science (ian.umces.edu/symbols/ accessed on 25 September 2023).

Seaweed can be broadly categorized into three main types, including brown, red, and green seaweed under the phylum groups of Phaeophyceae, Rhodophyta, and Chlorophyta, respectively. Within these phyla, there are a multitude of morphologies. Figure 1 depicts the various types of seaweeds. Seaweeds have evolved to either be attached to hard substrates, such as rocks, with the help of special root-like organs known as holdfasts, or be free-floating [25]. As highlighted above, seaweeds are a good source of essential nutrients such as proteins, vitamins, minerals, and bioactive compounds. Notably, the composition may vary depending on the season, water depth, salinity, and geography. The groups of seaweed and their nutritional composition are discussed below.

### 2.1. Brown Seaweed

Brown seaweed, which includes the structurally complex forms of kelp, is the most common type of seaweed found in coastal areas around the world. Brown seaweed gets its name from its characteristic brown or olive-green color, which is due to the presence of a pigment called fucoxanthin [26]. Generally, the protein content of brown seaweed is comparatively lower than the other seaweed classes. Typically, brown seaweed ranges between 5 and 15% [27], considerably lower than values reported for red (10–47%) and green seaweed (9–26%) counterparts on a dry weight basis [28]. Consequently, the essential amino acid content is low in comparison. However, a wide range of soluble fibers, including alginates, fucans, and laminarins, are typically found in brown seaweed; however, their content varies with species [29]. For instance, a substantially higher level (63.88%) of dietary fiber has been observed in *Fucus spiralis* compared to *Spatoglossum scroederi* (Phaeophyceae) (4.28%) [30]. Schmid et al. [31] reported that total fatty acid levels varied substantially (0.6 to 7.8% of DW) between species, with the highest levels being found in brown seaweed (7.6% of DW), followed by green seaweed (3.9% of DW), with red seaweed (2.2% of DW) recording the lowest average concentrations. Airanthi et al. [32] studied three species of brown seaweed and found the highest n-3 PUFA content in *Undaria pinnatifida* (67.05 mg/g of DW), compared to *Sargassum horneri* (34.52 mg/g of DW), and *Stephanocystis hakodatensis* (formerly *Cystoseira hakodatensis*) (*Phaeophyceae*) (37.10 mg/g of DW). Moreover, saturated fatty acids (myristic acid, palmitolenic acid) are abundant in the lipid fraction of several brown seaweeds, such as *Laminaria* sp. and *Undaria pinnatifida* [29]. In addition to macronutrients, brown seaweed contains a host of minerals in high concentrations, including Na, K, Ca, and Mg, as well as Fe, Zn, Mn, and Cu in lower concentrations [33]. Brown seaweed also contain essential vitamins such as vitamin B, C, and E [27], whilst high levels of polyphenolic bioactive compounds, including fucol, fucophlorethol, fucodiphloroetol, and ergosterol, have also been shown to be present [34].

### 2.2. Red Seaweed

Red seaweed, also known as red algae, is a type of marine seaweed that belongs to the phylum Rhodophyta. They are referred to as ‘red seaweed’, a characteristic color, due to the presence of pigments called phycoerythrins. Red seaweed constitutes a diverse group of organisms that can be found in both freshwater and marine environments around the world [35]. Red seaweed contains a higher level of protein (10–47% of DW) in comparison to green (9–26% of DW) and brown seaweed (5–15% of DW) [36]. However, the protein, peptide, and amino acid concentrations of seaweed are controlled by a variety of factors, most notably by seasonal change [37]. For example, the highest protein content (10% of DW) was found in red seaweed (*Acanthophora muscoides*) collected in the summer, which was reduced to 9% during the winter [37]. The lipid content is comparatively lower in this group, ranging from 1 to 3% and 0.7 to 3% in DW in *Chondrus crispus* and *Palmaria palmata*, respectively, with apparently no significant differences between species or seasons [37,38]. In terms of fatty acid composition, however, red algae have high EPA contents (1.3–10.4% DW) compared to green seaweed (0.87–2.10%). Furthermore, the soluble fiber content is usually higher in red seaweed (15–22% DW), such as *Chondrus* sp. and *Porphyra* sp. [34]. Along with other minerals, the iodine level is comparatively higher in red seaweed, especially *Gracilaria* sp. [39]. Finally, water-soluble vitamins (vitamins B and C) and carotenoids, precursors of fat-soluble vitamin A [29], as well as several biologically active substances such as monoterpenes, diterpenes, acetogenins, and sesquiterpenes, are also present in red seaweed [40].

### 2.3. Green Seaweed

Green seaweed refers to a group of marine seaweed that belongs to the phylum Chlorophyta. They are commonly found in coastal areas and shallow waters around the world. Green seaweed comes in a variety of shapes, sizes, and colors, ranging from small filamentous forms to large leafy or tubular structures. They typically have a green color due to the presence of chlorophyll, a pigment that enables them to photosynthesize [38]. The main genus groups within Chlorophyta are *Ulva*, *Codium*, *Chaetomorpha*, and *Cladophora* [41]. When comparing the protein content, the levels in green seaweed are comparatively higher compared to brown seaweed but lower than that of red seaweed. Specifically, the protein content typically ranges from around 10% to 20% of dry weight, depending on the species. Among green seaweed species, *Ulva intestinalis* contains up to 19.5% protein during summer [42]. Fluctuations in protein content could be attributed to various external factors, such as water temperature, season, geographical location, weather, and processing conditions [42]. In terms of lipid content, *Ulva lactuca* has a comparable lipid content (1.3% DM) to brown seaweed [43], whereas the lipid contents in *Ulva australis* (formerly *U. pertusa*) and *U. intestinalis* are reportedly between 2.1 and 7.4% and 7.3 and 8.7% of DW, respectively [42]. *Ulva* is enriched with soluble and insoluble proteins, fibers, and a great source of heteropolysaccharides known as ulvan [33,44]. *Ulva* spp. are also a great source of vitamins, particularly ascorbic acid (vitamin C) [45].

**Figure 2:**
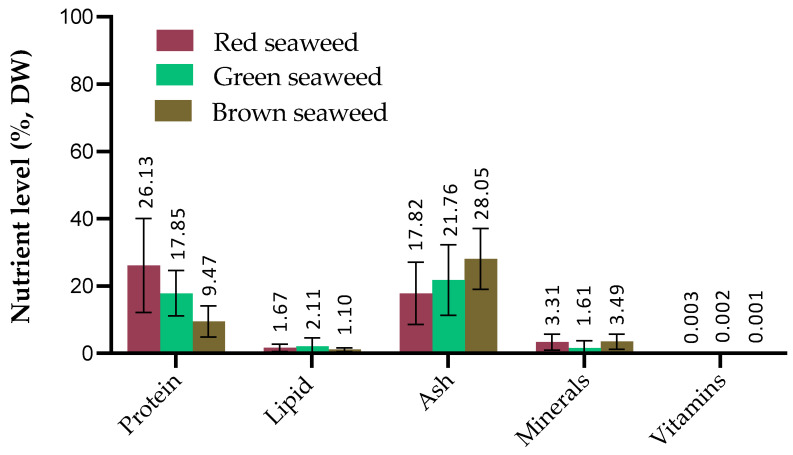
The nutritional composition (% of dry weight, DW) of various seaweed (red, green, brown) used in aquafeed formulations for fish. Approximately 30 research articles spanning 17 seaweed species were compiled for each value, demonstrating the average nutritional constituents of various seaweed species for the study.

The variations in nutritional composition of the three phyla groups of seaweed are depicted in Figure 2.

## 3. Seaweed-Based Functional Metabolites

In addition to basic nutrients, seaweed is the source of many bioactive compounds, including polysaccharides, polyphenols, and pigments. These active compounds in seaweed have led to numerous research studies exploring their potential applications in various fields, including fish nutrition. The common bioactive compounds found in seaweeds are stated below.

### 3.1. Polysaccharides

**Figure 3:**
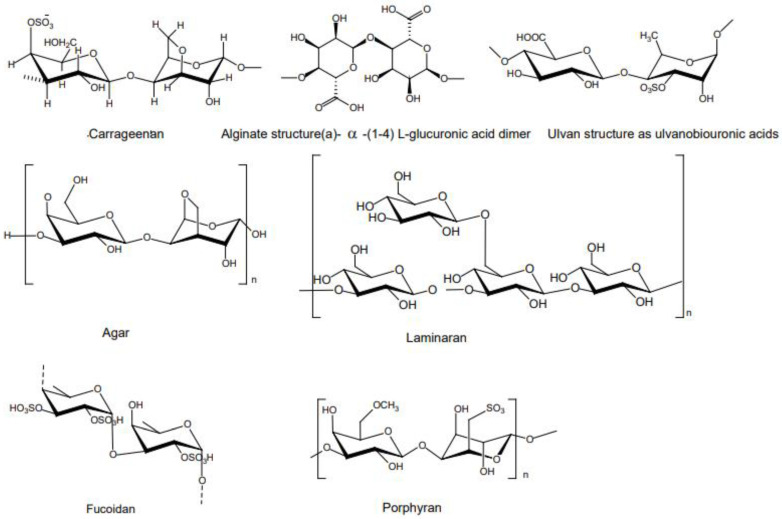
The chemical structures of various types of polysaccharides found in seaweed.

Polysaccharides, the most abundant macromolecule in seaweed, are categorized into two groups based on where they are found: cell-membrane polysaccharides and storage polysaccharides. With the exception of accumulating carbohydrates found in cell plastids, the majority of seaweed polysaccharides are cell-membrane polysaccharides. Agar, carrageenan, alginate, fucoidan, laminarin, ulvan, and xylan are among the structural and storage polysaccharides found in seaweed [46,47]. The chemical structures of available polysaccharides found in seaweed are presented in Figure 3. Agar and carrageenan are two of the most important polysaccharides generated from red seaweed. Some red seaweed also contains porphyrin, a sulfated polysaccharide that resembles agar and represents a high-quality dietary fiber. Several studies have indicated that sulphated polysaccharides (galactans and xylans) are abundant in green seaweed, whereas fucodian, laminarin, and alginic acid are abundant in brown seaweed, and xylans, carrageenans, galactan, and porphyrin are abundant in red seaweed [9,46,48,49]. The polysaccharide concentration in seaweed varies considerably, however, and has been reported to range between 4% and 76% of dry weight depending on the type of seaweed, species, location, and environmental factors [50]. Additionally, the specific content of these polysaccharides can be influenced by the processing methods used to extract them from the seaweed for commercial and industrial purposes [50]. Alginate is derived from brown seaweed, such as kelp and *Laminaria* species. Laminarin is generally found in *Laminaria*, *Ascophyllum*, *Fucus,* and *Undaria* sp. [51,52].

### 3.2. Phenolic Compounds

**Figure 4:**
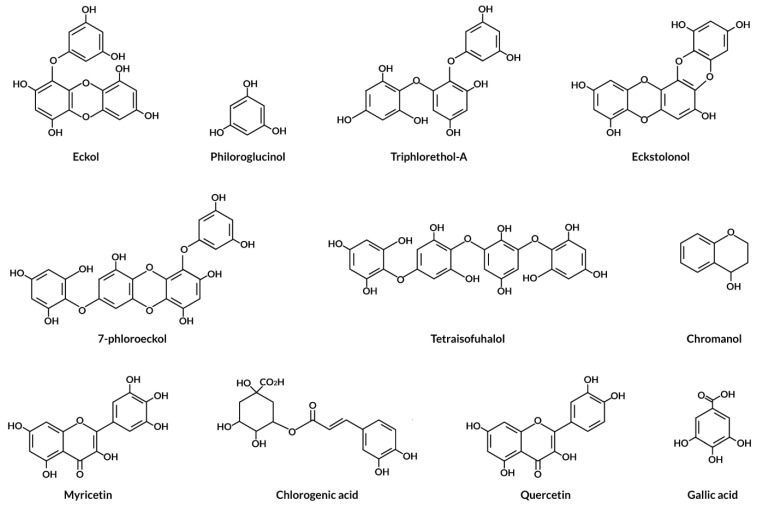
The chemical structures of various types of phenols found in seaweed.

Seaweed is a valuable source of polyphenolic compounds such as phlorotannins, bromophenols, flavonoids, and phenolic acids [53]. Bromophenols, phenolic acids, and flavonoids make up the majority of phenolic compounds found in green and red seaweed, while phlorotannins have mostly been identified in brown seaweed [53,54]. The chemical structures of available phenolics found in seaweed are displayed in Figure 4. Aqueous extracts of brown seaweed (*Halopteris scoparia* (formerly *Stypocaulon scoparium*) may contain considerable amounts of phenolic acids and flavonoids, 90 mg/100 g DW of gallic acid, followed by catechin and epicatechin (6–7 mg/100 g DW) [55]. Yumiko et al. [56] investigated the flavonoid distribution in methanolic extracts of 27 Japanese seaweed species (6 green, 11 brown, and 10 red seaweeds), finding that red seaweeds had a higher amount of flavonoids than green and brown seaweeds. Hesperidin was discovered in all red seaweeds (626–119,000 µg/g), as well as some green and brown seaweeds [56]. Catechol was found in all green and red seaweeds (1660–77,700 µg/g) but not in the majority of brown seaweed [56]. Rutin and caffeic acids were found in all three groups but were most abundant in red seaweed (23200–4000 µg/g) [56]. Quercitrin and myricetin were rarely discovered in brown and red seaweeds (202–466 µg/g), although Morin was identified in minor amounts in all seaweed samples (257–3730 µg/g) [56]. Further, Onofrejova et al. [57] identified twelve phenolic acids in extracts of red seaweed (*Pyropia tenera*) and brown seaweed (*Undaria pinnatifida*), namely hydroxybenzoic acid (salicylic acid, 2,3-dihydroxybenzoic, p-hydroxybenzoic, protocatechuic), hydroxycinnamic acid (p-coumaric, caffeic, chlorogenic), and hydroxybenzaldehydes (3,4-dihydroxybenzaldehyde, p-hydroxybenzaldehyde). Phlorotannin is a heterogeneous group of polymeric compounds found significantly in brown seaweed that includes Laminariales (*Ecklonia* spp. and *Eisenia* spp.), Fucaceae (*Ascophyllum nodosum* and *Fucus vesiculosus*), and *Sargassaceae* families [58]. Phlorotannin concentrations of up to 15% in dry weight (DW) have been observed in brown seaweeds, with *Fucus* sp. being reported as making up 12% of DW and *A. nodosum* being reported as making up 14% of DW [34].

### 3.3. Pigments

**Figure 5:**
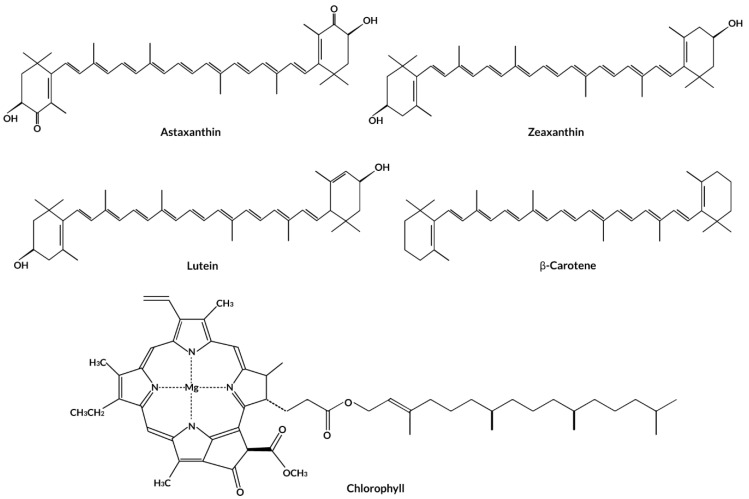
The chemical structures of various types of pigments found in seaweed.

As briefly mentioned, chlorophylls, carotenoids, and phycobiliproteins are the three major groups of seaweed pigments. Seaweeds are divided into three groups depending on pigment content: seaweed that is rich in chlorophylls *a* or *b* appears green, whereas seaweed that is rich in fucoxanthin (carotenoid) appears greenish-brown, and seaweed that is rich in chlorophylls *a*, *c*, or *d* and phycobilins appear red [59]. In general, chlorophylls are greenish, non-polar pigments that contain porphyrin or hydroporphyrin rings centrally bound to a magnesium atom, which are found in all autotrophic seaweeds. The level of chlorophyll-*a* ranged between 565 and 2000 mg/kg DW in brown seaweed [60]. Carotenoids are lipophilic, linear polyenes in two categories: (i) carotenoids and lycopene and (ii) xanthophylls (e.g., antheraxanthin, zeaxanthin, lutein, fucoxanthin, violaxanthin). Carotenoids, which have a characteristic linear C40 chain, contain up to 11 conjugated bonds (allenic bonds) that may engage in antioxidant activities via the transfer of singlet oxygen excess energy in the long central allenic chain. Furthermore, as demonstrated in astaxanthin and fucoxanthin, the allenic bonds and other functional groups in the structure’s terminal rings may react with free radicals, adding to their antioxidant capacity. The chemical structures of some common pigments found in seaweed are presented in Figure 5. A study by Balbasubramaniam et al. [61] found that carotenoids in red seaweed (*Eucheuma denticulatum*) consisted of lutein, zeaxanthin, β-cryptoxanthin, and β-carotene, with lutein present as the major content in this class of seaweed with a concentration of 87.7 mg/100 g DW. Fucoxanthin (2740 mg/100 g DW) is the predominant carotenoid found in the brown seaweed (*Sargassum polycystum*), whereas β-carotene and canthaxanthin (14.6 mg/100 g and 19.5 mg/100 g DW, respectively) were the highest carotenoid contents in green seaweed (*Caulerpa lentillifera*). Phycobiliproteins, on the other hand, are a group of water-soluble fluorescent compounds composed of proteins covalently bound to linear tetrapyrroles known as phycobilins, and they can represent up to 40–50% of the total cellular proteins in red seaweed [62].

Some of the notable bioactive compounds and their concentrations reported in various seaweeds are presented in Table 1.

## 4. Most Commonly Utilized Seaweeds in Aquafeed

In 2019, seaweed cultivation accounted for nearly 30% of total aquaculture production. Currently, red seaweed and brown seaweed are the second and third most produced species groups in aquaculture globally, behind only “Carps, barbels and other cyprinids” [77]. Currently, a variety of seaweed species are used as supplemental fish feed ingredients as sources of amino acids, fatty acids, antioxidants, vitamins, minerals, pigments, and polysaccharides. A summary of the specific types of seaweed that are most commonly used in aquafeed for fish is listed in Table 2.

## 5. The Role of Seaweed in Aquaculture Production

Seaweed is a potential source of essential nutrients that can be used as a sustainable and cost-effective supplement to traditionally used aquafeed ingredients for fish. Incorporating seaweed into the diets of farmed fish can improve their growth, health, and resistance against invading pathogens, thereby improving disease resistance. Since there is a dearth of knowledge regarding the use of seaweed functional metabolites in aquafeed for fish, the studies discussed here are mostly on the use of seaweed and seaweed-based extracts in fish nutrition. An overview of some key effects on fish production when seaweed is included in aquafeed is presented below.

### 5.1. Growth Performance and Feed Utilization

#### 5.1.1. Growth Performance

**Figure 6:**
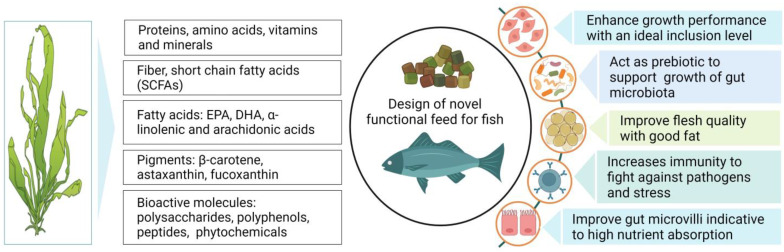
Schematic diagram demonstrating the composition of seaweed and the potential impacts of its addition on growth and health performance of fish.

The effectiveness of seaweed as a feed additive varies greatly depending on the nutritional profile and the species-specific feeding nature of fish [78,79]. In general, low dietary inclusion of seaweed, up to 10%, has been shown to impart significant improvements in growth, feed utilization, and the assimilation of essential nutrients [80,81]. The dietary supplementation of Laminaria sp. with levels of 3 and 10% has been shown to significantly enhance the daily feed intake and weight gain in Atlantic salmon [82]. Likewise, Sony et al. [83] found that dietary supplementation of fucoidan, a polysaccharide derived from brown algae (*Cladosiphon okamuranus*), at a level of 0.4%, significantly improved the growth performance of juvenile red sea bream (*Pagrus major*). In contrast, the inclusion of red seaweed (*Porphyra dioica*) at up to 10% of the diet did not affect the growth, whilst a 15% inclusion caused a significant growth reduction in rainbow trout (*Oncorhynchus mykiss*) [12]. Similarly, a 6% dietary provision of *Gracilaria pygmaea* enhanced the growth performance of *O. mykiss*, while a 12% inclusion evoked negative impacts on growth [84]. Moreover, Soler-vila et al. [17] reported that 10% dietary red alga (*Porphyra dioica*) exhibited no negative impacts on the growth of rainbow trout, while 15% inclusion showed negative results compared to the control. Overall, these observations indicate that seaweed, when incorporated at an appropriate inclusion level, can either significantly improve or maintain growth performance at similar levels to non-seaweed diets, whereas higher inclusion levels can negatively impact the growth and health status of fish. Notably, the higher growth observed with seaweed-supplemented diets is likely attributable to elevated concentrations of bioactive compounds (phytonutrients, i.e., essential vitamins and minerals) [85,86] that play vital roles in the enhanced assimilation of dietary nutrients in fish [87,88]. Interestingly, it has also been speculated that seaweed contains a wide range of polysaccharides and oligosaccharides that act as prebiotics, which promote the activity of beneficial bacteria and thus enhance the digestion and absorption of essential nutrients, subsequently improving growth performance in fish [89]. On the other hand, reduced growth performance at higher inclusion levels (>10%) of seaweed in aquafeed may potentially be caused by the presence of substantial concentrations of antinutritional substances emanating from the seaweed that exerts various toxicity effects and restricts the absorption of essential nutrients [89,90]. For instance, protease inhibitors are found in many plant-based feeds, including seaweed. These are molecules that inhibit the activity of protease enzymes, which are responsible for breaking down proteins into smaller peptides and amino acids. When fish are fed a higher quantity of seaweed, they can bind to proteolytic enzymes and interfere with the normal digestive process by inhibiting the activity of digestive enzymes. This can lead to incomplete protein digestion, reduced nutrient absorption, and overall poor performance in fish [91]. Further, instances of growth reduction may also be attributable to the polysaccharide content in seaweed, which may influence the rapid transition of feed through the digestive tract, in turn causing enhanced feed uptake while lowering the absorption of nutrients [92,93]. Therefore, the removal or breakdown of these complex carbohydrates and antinutritional factors in seaweed via the incorporation of novel processing technologies may permit higher nutrient absorption efficiency and fish growth. The effects of seaweed supplementation on the growth performance of various fish species are presented in Table 3. A snapshot of some of the major effects of seaweed supplementation in aquafeed on fish performance is depicted in Figure 6.

#### 5.1.2. Feed Conversion Ratio (FCR)

FCR remains a fundamental metric to assess feed efficiency in fish, where a lower value represents an improved conversion of feed to fish biomass gain. The reliance on this metric stems from the fact that feed inputs are a major cost for intensive aquaculture operations. Several feed additives, including seaweed, have been incorporated into aquafeed to improve the FCR. Several studies reported that dietary seaweed inclusion resulted in a lower FCR in Nile tilapia [21,94], *Salmo salar* [82], *Pagrus major* [83], *Acanthopagrus schlegelii* [95], and *Labeo rohita* [96]. Improvements to FCR could be partly due to the presence of various bioactive compounds (carotenoids, polysaccharides, amino acids, and fatty acids) that significantly improve the palatability and, consequently, intake of feed, hence improving feed utilization [97]. Bioactive substances have been shown to stimulate the secretion of several enzymes (amylase, lipase, and protease) that are known to enhance the digestion of essential nutrients as well as their assimilation into fish tissues [98]. Likewise, improved FCR could result from the activities of the polysaccharides of seaweed that slow the passage of feed through the digestive tract, which ensures greater nutrient assimilation and bioavailability [84,99]. In addition, seaweed as a source of prebiotics may enhance the growth of beneficial bacteria in the gut, significantly improving digestibility and feed efficiency [100]. However, contrasting findings to those articulated above have been reported, with several studies indicating that dietary seaweed did not significantly affect feed utilization across a range of fish species, including seabass (*Dicentrarchus labrax*), Senegalese sole (*Solea senegalensis*), and gilthead seabream (*Sparus aurata*) [81,101,102]. Notably, high levels of seaweed in aquafeed may reduce the palatability of fish [103]. Moreover, these variations may be exhibited by the feed composition, physiology of fish species, size of the species, environmental quality, as well as the dietary inclusion level of the seaweed.

#### 5.1.3. Feed Palatability

The palatability of aquafeed is one of the most crucial factors influencing the consumption of feeds by farmed species [104]. A reduction in palatability may lead to an increase in feed wastage, resulting in reduced fish production and negatively impacting the profitability of aquaculture operations. On the contrary, highly palatable feeds increase feed consumption and effectiveness, generally resulting in better fish growth, assuming the nutritional requirements of the species are being met (Table 3). However, the palatability of an aquafeed is largely influenced by the nutrient composition of its ingredients as well as feed processing techniques, nutrient digestibility, water stability, and species-specific nutritional requirements and physiology of fish [105]. The application of several plant-origin protein sources, including those emanating from algal species, to enhance the palatability of fish feed has attracted considerable research attention in recent times. Kamunde et al. [82] reported that Atlantic salmon consumed more feed when brown seaweed (*Laminaria* sp.) was included in the diet in comparison to a seaweed-free control feed. Similarly, greater consumption of an *Ulva* sp.-based diet was reported in seabream (*S. aurata*) [106] and sea urchin (*Tripneustes gratilla)* [107]. This higher feed response may be attributable to several bioactive compounds such as dimethyl-beta-propionthein, dimethyl sulfonyl propionate, amino acids, and peptides that enhance the attraction of a feed to the farmed fish species and, in turn, increase feed consumption [108,109]. In addition, the inclusion of seaweed can improve the overall physical structure of an aquafeed, including integrity, texture, and water stability, all of which are factors that may contribute to increased feed intake [110]. Furthermore, the volatile organic compounds emitted by seaweed [111] can contribute to the aroma and flavor of the feed, making it more attractive and palatable to fish. This could potentially lead to increased feed intake and improved growth rates. However, the application of seaweed in aquafeed should be carefully considered as high inclusion levels have been reported to reduce feed palatability and feed consumption, in turn negatively impacting the growth and health status of fish [90].

#### 5.1.4. Feed Digestibility

The efficiency of an aquafeed is highly dependent on the digestibility of its constituent feed ingredients (Table 3). The incorporation of ingredients with a high digestible value will minimize feed wastage and maximize feed utilization, thus improving growth performance. It has been reported that apparent nutrient digestibility coefficients (ADC) of protein, lipid, and energy were not changed when up to 20% of *Ulva* sp. was included in diets for Nile tilapia [112]. Pereira et al. [113] revealed that the digestibility of *Ulva* meal in diets for Nile tilapia was higher in comparison to diets containing *Gracilaria* or *Porphyra*. Contrarily, Soler-vila et al. [12] found that up to 15% inclusion of *Porphyra dioica* did not result in significant alterations in comparison to a control diet in rainbow trout. However, Azaza et al. [92] reported that a 10% replacement of soybean meal with *Ulva rigida* decreased the ADC of protein in Nile tilapia from 87% to 82%. Notably, the effect of seaweed inclusion on nutrient ADC appears to be dependent on the type of seaweed itself, the nature of fish species, feed composition, and the degree of inclusion of the examined seaweed and, thus, the protein source being substituted. Different seaweed species exhibit varying effects on nutrient digestibility in different fish species, which can largely be explained by feeding habits and gut morphology, which determines the capacity for digestion and absorption of the nutrients contained in seaweed [114]. Most herbivorous and omnivorous fish species exhibit a higher level of amylase activity for the enhanced breakdown of the carbohydrates provided by dietary seaweed inclusion [80,115]. Importantly, carnivorous fish species have a reduced ability to break down complex seaweed polysaccharides due to a lack or limited amount of these enzymes [116]. As such, coupling digestive enzyme activity with the morphology of the gastrointestinal tract, the capacity for dietary seaweed incorporation into aquafeeds will likely be dictated by trophic level, where it stands to reason that herbivorous and omnivorous species will have a much higher tolerance to dietary seaweed than their carnivorous counterparts.

**Table 3:** Effects of seaweed and seaweed-based functional metabolites on growth, feed utilization, immunity, and disease resistance in farmed fish (studied parameters were compared to control—0% FM diet).

Seaweed and Derivatives	Fish Species	Applied Levels	Effective Level	Trial Period (Day)	Response	Reference
*Sargassum portieranum* (Phaeophyceae)	*Oreochromis niloticus*	5 and 10%	10%	84	SW inclusion resulted in significant growth enhancement	[117]
*Grateloupia acuminata* and *G*. *doryphora* (Rhodophyta)	*O. niloticus*	0.1, 0.25, 0.5, and 1.0%	0.5 and 1.0%	60	Growth and digestibility increased compared to control	[118]
Polyphenols from *Eisenia arborea* (Phaeophyceae)	*Haliotis fulgens*	13.9 and 33.3 mg/g	-	12	Polyphenol reduction in feed promoted feed attractiveness and consumption	[119]
Fucoidan from *Fucus vesiculosus* (Phaeophyceae)	*Danio rerio*	100 μg/mL	-	5	Fucoidan reduced NO and ROS accumulation in *D. rerio* larvae, which indicated therapeutic role of fucodian against inflammatory disorder	[120]
Fucoidan from *Saccharina japonica* (Phaeophyceae)	*Clarias gariepinus*	0.04 and 0.06%	-	21	Dietary fucodian significantly enhanced the phagocytic activity, serum lysozyme, and bactericidal activity	[121]
Fucodian from *Cladosiphon okamuranus* (Phaeophyceae)	*Pagrus major*	0.4%	-	56	Fucoidan supplementation showed nonsignificant improvement in feed utilization. Catalase activity is significantly influenced by fucodian	[122]
Fucodian from *Undaria pinnatifida* (Phaeophyceae)	*Marsupenaeus japonicus*	0.01, 0.05, and 0.10%	0.05%	56	0.05% fucodian supplementation remarkably increased the growth and immune performances	[123]
Fucodian from *Undaria pinnatifida* (Phaeophyceae)	*Lates calcarifer*	0.5 and 1.0%	1.0%	52	1% fucoidan inclusion diet exhibited enhanced growth	[124]
*Gracilaria persica* (Rhodophyta)	*Acipenser persicus*	0.25, 0.5, and 1.0%	0.5 and 1.0%	56	No significant improvement in growth due to SW provision	[125]
Mixture of *Ulva lactuca* (Chlorophyta), *Jania rubens,* and *Pterocladia capillacea* (Rhodophyta)	*O. niloticus*	0.5, 1, 1.5, and 2.0%	2.0%	70	Growth promoted at 2% dietary SW	[94]
*Gracilaria* sp. (Rhodophyta), *Ulva* sp. (Chlorophyta), or *Fucus* sp. (Phaeophyceae)	*D. labrax*	2.5 and 7.5%	7.5%	49	Immunity and antioxidant status improved at 7.5% SW inclusion compared to control	[81]
*Laminaria* sp. (Phaeophyceae)	*S. salar*	3, 6, and 10%	10%	30	Growth and immune status developed at 10% SW inclusion	[82]
*Gracilaria pygmaea* (Rhodophyta)	*O. mykiss*	3, 6, 9, and 12%	9%	56	Growth improved at 9% SW, while it was reduced at 12% SW level	[84]
Fucodian from *Cladosiphon okamuranus* (Phaeophyceae)	*P. major*	0.05, 0.1, 0.2, 0.4, and 0.8%	0.4%	60	Growth promoted at 0.4% dietary SW. Enhanced immune response and disease resistance at 0.3–0.4% SW	[83]
*Ulva lactuca* (Chlorophyta) Jania rubens and Pterocladia capillacea (Rhodophyta)	*Pangasianodon hypophthalmus*	1, 2, and 3%	2%	60	SW at a level of 2% improved the growth and resistance against *Aeromonous. hydrophila* infection.	[126]
*Pelvetia canaliculata* (Phaeophyceae)	*Sparus aurata*	1, 5, and 10%	-	56	SW inclusion produced no changes in proximate composition and the fatty acid profile of fish when compared to control	[127]
*Gracilariopsis lemaneiformis* (Rhodophyta)	*Pagrosomus major*	3, 6, 9, 12, and 15%	3%	56	Growth improved at 3% SW. Liver glycogen and hepatic AST were significantly higher in supplemented group	[128]
*Sargassum wightii* (Phaeophyceae)	*L. rohita*	2%	-	45	Growth promoted by dietary SW without compromising its immune-modulating effects	[96]
*Ulva prolifera* (formerly *Enteromorpha prolifera*) (Chlorophyta)	*O. mossambicus* × *O. niloticus*	1, 2, 3, 4, and 5%	5%	49	Growth was enhanced by dietary *U. prolifera.* SOD, LYZ, acid phosphatase and alkaline phosphatase activities were enhanced	[129]
*Gracilaria arcuata* (Rhodophyta)	*O. niloticus*	20, 40, and 60%	20%	84	Growth and feed utilization improved at 20% SW	[130]
*Gracilariopsis persica*, *Hypnea flagelliformis* (Rhodophyta), and *Sargassum boveanum* (Phaeophyceae)	*O. mykiss*	5 and 10%	-	83	Serum LYZ, SOD, and CAT activity increased by SW provision	[22]
*Gracilaria pulvinata* (Rhodophyta)	*Lates calcarifer*	3, 6, and 9%	3%	40	No growth retardation up to 3% SW. Serum LYZ activitywas significantly enhanced at 3% supplementation, while ACH50 was lowered at 9% SW	[131]
*Ulva rigida* (Chlorophyta) and *Undaria pinnatifida* (Phaeophyceae)	*Solea senegalensis*	10%	-	150	Growth retardation observed in growing stage for *Undaria*-based diet	[101]
Mixture of *Gracilaria* sp. (Rhodophyta)*, Ulva* sp. (Chlorophyta)*,* and *Fucus* sp. (Phaeophyceae)	*D. labrax*	7.5%	-	63	Did not mitigate negative effects of environmental oscillations on growth and immunity by dietary SW	[132]
*Ulva* sp. (Chlorophyta)	*Argyrosomus japonicus*	5, 10, and 20%	5%	63	Growth and feed utility increased at 5% SW	[133]
*Ulva lactuca* (Chlorophyta)	*S. aurata*	2.6 and 7.8%, 14.6 and 29.1%	-	140	No growth retardation observed by dietary SW	[134]
*Gracilaria pygmaea* (Rhodophyta)	*O. mykiss*	3, 6, 9, and 12%	6%	49	Growth was enhanced at 6% SW	[135]
*Gracilaria* sp. (Rhodophyta) and *Alaria* sp. (Phaeophyceae)	*A. regius*	5%	-	69	No growth retardation by SW addition. Lipid peroxidation lowered	[136]
*Gracilariopsis lemaneiformis* (Rhodophyta) and *Sargassum horneri* (Phaeophyceae)	*Lutjanus stellatus*	5, 10, 15, and 20%	15%	60	Growth retardation at 20% SW	[137]
*Taonia atomaria* (Phaeophyceae)	*O. niloticus*	5, 10, and 15%	5%	84	Significant growth improvement by SW inclusion	[138]
*Palmaria palmata* (Rhodophyta)	*S. salar*	5, 10, and 15%	-	98	ALT activity significantly decreased with no effects on LYZ or ACH50 activity	[139]
*Ulva lactuca* (Chlorophyta)	*Lutjanus stellatus*	5, 10, 15, and 20%	5%	60	Growth promoted at 5% SW	[140]
*Sargassum angustifolium* (Phaeophyceae)	*O. mykiss*	0.005, 0.01, 0.02, and 0.04%	-	56	Immune status and lower mortality against *Yersinia rukeri* by dietary SW	[141]
*Gracilaria* sp. (Rhodophyta)	*D. labrax*	0.5 and 4.5%	-	42	ACH50 activity was enhanced, while no effect was observed on LYZ and PO activity by SW inclusion	[142]
*Sargassum dentifolium* (Phaeophyceae)	*O. mossambicus* × *O. niloticus*	1, 2, and 3%	3%	84	Significantly increased GOT and triglycerides level, while no impact was noticed for total plasma protein, albumin, and globulin	[143]
*Saccharina latissimi* (Phaeophyceae)	*O. mykiss*	1, 2, and 4%	1 and 2%	84	Significantly downregulated the expression of stress marker (gpx1b2)	[144]
*Ulva prolifera*, *Ulva australis* (formerly *U. pertusa*) (Chlorophyta), or *G. lemaneiformis* (Rhodophyta)	*Siganus canaliculatus*	12%	-	70	LYZ, dismutase, and acid phosphatase were significantly enhanced. Enhanced resistance against *Vibrio parahaemolyticus*	[145]
*Ulva* sp. (Chlorophyta)	*O. niloticus*	5 and 10%	10%	68	Significantly enhanced ACH50 activity, while no effects were observed in the cases of LYZ and PO activity	[146]
*Padina gymnospora* (Phaeophyceae)	*Cyprinus carpio*	0.01, 0.1, or 1%	-	21	Remarkably improved serum LYZ, MPO, and antibody responses	[147]
*Enteromorpha intestinalis* (Chlorophyta)	*O. niloticus*	10, 20, 30, and 40%	20%	42	Significantly improved growth performance at 20% inclusion level	[148]
*Sargassum fusiformis* (formerly *Hizikia fusiformis*) (Phaeophyceae)	*Paralichthys olivaceus*	0, 0.5, and 1%	-	84	Significantly upgraded the immune status of fish by raising the level of hepatic IL-2 and IL-6	[149]
*S. fusiforme* and *Ecklonia cava* (Phaeophyceae)	*Paralichthys olivaceus*	6%	-	42	Hb level and RBC count were significantly elevated. Exhibited higher resistance against *Edwardsiella tarda* challenge	[150]
*Ulva lactuca* (Chlorophyta) and *Pterocladia capillacea* (Rhodophyta)	*D. labrax*	5, 10, and 15%	-	56	*P*. *capillacea* exhibited high-stress resistance capacity compared to *U*. *lactuca*	[14]
*Eucheuma denticulatum* (Rhodophyta) and *Sargassum fulvellum* (Phaeophyceae)	*P. olivaceus*	3 and 6%	6%	56	Significantly lowered the level of blood cholesterol and triglycerides. Serum LYZ activity was significantly enhanced	[151]
*Sargassum whitti* (Phaeophyceae)	*M. cephalus*	0.5, 1.0, and 1.5.0%	-		WBC, LYZ, and RBC significantly elevated in seaweed-supplemented groups. Mortality rate decreased after exposure to *Pseudomonas fluorescence*	[152]
*Gracilariopsis lemaneiformis* (Rhodophyta)	*Siganus canaliculatus*	33%	-	56	LYZ and ACH50 activity was remarkably enhanced in the group provided seaweed	[153]
*Ecklonia cava* (Phaeophyceae)	*P. olivaceus*	2, 4, and 6%	-	42	Serum LYZ, MPO, and NBT activities were significantly increased	[154]
*Macrocystis pyrifera* (Phaeophyceae) and *Chondrus crispus* (Rhodophyta)	*Epinephelus coicoides*	0.001, 0.002, and 0.003%	-	5	Significantly enhanced RBC, SOD, and phagocytic activity. Exhibited resistance against *V. alginolyticus*	[155]
*Sargassum fusiforme* (formerly *Hizikia fusiformis*) (Phaeophyceae)	*P. olivaceus*	2, 4, and 6%	-	56	Phagocyte activity was elevated with the increase of *S. fusiforme* in diet. Improved resistance to *Streptococcus iniae*	[156]
*Ulva lactuca* (Chlorophyta) *Pterocladia capillacea* (Rhodophyta)	*S. aurata*	5, 10, and 15%	5 and 10%	56	Enhanced stress response ability	[157]

Note: SW—seaweed; FM—fish meal; WG—weight gain; FCR—feed conversion ratio; PER—protein efficiency ratio; FE—feed efficiency; ADC—apparent daily co-efficient; FI—feed intake; PM—poultry meal; GOT—glutamic-acid-oxyl acetic-acid-transaminase; SOD—superoxide dismutase; GP—glutathione peroxidase; PO—phenoloxidase; LYZ—lysozyme activity; CAT—catalase activity; ACH50—alternative complement activity; MPO—myeloperoxidase; NBT—nitroblue tetrazolium; RBC—red blood cell; WBC— white blood cells; Hb—haemoglobin; AST—aspartate transaminase; ALT—alanine transaminase; ROS—reactive oxygen species; NO—nitric oxide; - — not identified.

### 5.2. Immune Status, Antioxidant Response, and Gut Health in Fish

#### 5.2.1. Immunity and Disease Resistance

Disease remains a great threat to the intensive aquaculture sector, hindering industry growth and potentially leading to huge economic losses [158]. The main purpose of intensive aquaculture systems is to ensure maximum production within a limited culture period. In some circumstances, on-farm efficiency may be improved by operating at high stocking densities, potentially causing a deterioration of water quality to the detriment of fish health through the suppression of the immune system and the disruption of antioxidant defense mechanisms. To address these challenges, different types of drugs are commonly used for the treatment of disease [159]. However, their indiscriminate use has led to growing concerns for the surrounding environment as well as public health via the direct consumption of treated farmed fish or through the consumption of wild fishes located in the areas surrounding the treated aquaculture farm [160,161]. Furthermore, the rapid application of antibiotics may give rise to antibiotic-resistant bacteria [162] that significantly reduce the efficacy of antibiotics in controlling diseases. Therefore, it is of the utmost importance to seek possible environmentally friendly prophylactic measures. The application of seaweed-based feed ingredients as immunostimulants to strengthen the immune status of fish is viewed as a suitable alternative. An overview of the role of seaweeds as immunostimulators in fish is presented in Table 3. Mendonca et al. [163] revealed that 5% dietary *Gracilaria domingensis* (Rhodophyta) improved the immune response of juvenile mullet (*Mugil liza*) by modulating the activity of glycoproteins CD3 and CD4. Similarly, 6% dietary *S. hornei* promoted the antioxidant profile and immune capacity of black sea bream (*Acanthopagrus schelegelii*) [95]. Likewise, the green seaweed (*Ulva lactuca* (formerly *Ulva fasciata*) (Chlorophyta)) greatly improved the innate immune response of Nile tilapia through the modulation of lysozyme and phagocytic activity, as well as total WBC count and overall antioxidant status [164]. Similarly, the addition of red microalga (*Porphyridium* sp.) in the diet of pompano (*Trachinotus ovatus*) significantly upregulated the levels of mRNA c-type lysozyme and complement C4 while downregulating the mRNA heat shock protein (HSP70), thus playing an important role in the improvement of non-specific immune responses [165]. On the contrary, besides the positive role of seaweeds and their derivatives, the review of Thepot et al. [6] revealed no significant impacts in stimulating growth and immune responses in fish. This may be attributed to the very short (14-day) feeding trial that was likely insufficient to exhibit a significant immune response, given most immunostimulating compounds are reported after longer (49-day) feeding trials [166].

Alternatively, Wang et al. [167] observed that dietary *Sargassum horneri* (Phaeophyceae) did not significantly alter the lysozyme activity of juvenile turbot (*Scophthalmus maximus*) in comparison to the control. This variation may be caused by genetically driven species-specific responses of fish to the associated seaweed species. In addition, diseases in the aquaculture sector cost the industry in excess of USD 6 billion each year [168]. Fish are often subjected to various pathogenic organisms, which can lead to several diseases that negatively impact their health status and growth performance. In this situation, the utilization of seaweed and its bioactive substances as suitable alternative strategies can enhance both the cellular and humoral immune response towards disease resistance. Zeraatpisheh et al. [141] reported that the supplementation of *Sargassum angustifolium* (Phaeophyceae) in the diet of rainbow trout positively modulated fish immunity via increased hemoglobin (Hb), hematocrit (Hct), red blood cells (RBC), white blood cells (WBC), and phagocytes. Likewise, elevated lysozyme activity (LYZ), the expression of immune-related genes (e.g., il-1β, tnf-α), and the modulation of resistance to pathogenic bacterial infection from *Yersinia rukeri* (*Pseudomonas fluorescens*) were also reported. Several recent studies have also demonstrated that dietary provision of *Sargassum ilicifolium* (Phaeophyceae), *Gracilaria* sp. (Rhodophyta), *Ulva ohnoi*, and *Sarcodia suiae* (Rhodophyta) improved the nonspecific immunity and disease resistance of great sturgeon (*Huso huso*) [169], *Dicentrarchus labrax* [142], *Solea senegalensis* [170], and *O. niloticus* [171], respectively. In addition, Wang et al. [167] revealed that dietary *Sargassum horneri* (Phaeophyceae) significantly enhanced the non-specific immunity of juvenile turbot (*S. maximus*) and its disease resistance against *Edwardsiella tarda*.

#### 5.2.2. Antioxidant Response

Seaweed and its extracts exhibit excellent antioxidant and immunomodulatory properties [172,173]. Inoculation of seaweed extracts (sodium alginate and carrageenan from *Macrocystis pyrifera* and *Chondrus crispus*) in grouper (*Epinephelus coicoides*) resulted in a significant enhancement of respiratory burst; superoxide dismutase and phagocytic activities that are the key indicators of antioxidant status [155]. Peixoto et al. [18] reported that a 2.5% inclusion of dietary *Gracilaria* spp. significantly promoted glutathione peroxidase (GPx) activity in European seabass, which may be attributed to the elevated levels of selenium found in *Gracilaria* spp. that contribute to increased GPx production [174,175]. In addition to enhanced lipid peroxidation, increases in glutathione reductase and glutathione s-transferase have been reported as a result of dietary *Gracilaria* inclusion [81], clearly indicating the influence of *Gracilaria* sp. inclusion with respect to the modulation of fish antioxidant profile and the stress status of fish. Moreover, in Atlantic salmon, the supplementation of *Laminaria* sp. not only significantly increased the total plasma antioxidant status but also activated several mitochondrial antioxidant enzymes, including catalase, superoxide dismutase (SOD), and total glutathione level [82]. Similar results have also been reported in rainbow trout fed diets supplemented with *Gracilaria pygmaea* [84]. In addition, the dietary provision of either whole brown seaweed (*Ascophyllum nodosum*) or its extract significantly modulated the serum antioxidant profile, lowered lipid peroxidation, and enhanced the activity of SOD in ruminants [176,177]. All of these findings clearly highlight the potential role of seaweeds in modulating the antioxidant status of fishes either directly via an elevation of antioxidant substances or via an improvement to the functioning of antioxidant defense mechanisms.

### 5.3. Intestinal Morphology

The intestines of fish are important organs that play a vital role in fish’s immune statuses [178]. The morphological structure of the gastrointestinal tract (GIT) acts as an important indicator of the nutritional status and physiological state of fish [179]. Several studies have reported that dietary seaweeds, such as *Sargassum dentifolium* (*S. ilicifolium*) (Phaeophyceae), do not alter the normal intestinal tissue structure (e.g., enterocyte length and width and thickness of villi) [143,180], indicating its suitability as a feed ingredient. Dietary seaweed supplementation has also been shown to improve the intestinal epithelial mucosa, indicating an enhanced immune capacity of fish [91]. The immune activities of fish intestines are greatly dependent on the condition of the associated intestinal barriers that primarily consist of epithelial cells [181]. These epithelial cells assist in the production of IgA through the activation of T cells and B cells that play a defensive role against various antigens [181]. Dietary *Laminaria digitata* (Phaeophyceae) and *Gracilaria gracilis* (Rhodophyta) significantly boosted the intestinal acid goblet cells of mullet (*Liza ramada*) [182] and European seabass (*D. labrax*) [183,184], which perform key roles in intestinal immune activity. Goblet cells act as a protector of intestinal barriers through the production and secretion of mucus and antimicrobial proteins (chemokines and cytokines), enhancing the local immune response of the intestine [185]. Yu et al. [103] demonstrated that *Gracilariopsis lemaneiformis* provision in the diet of *Litopenaeus vannamei* increased the villi length of the intestine, which significantly improved the absorption capacity of several nutrients. Similarly, the provision of 1 to 3% *Undaria pinnatifida* in diets for shrimp (*Penaeus monodon*) significantly enhanced the length of intestinal fold when compared to the control [186]. On the contrary, 10% *Gracilaria* sp. supplementation caused lower villi length and diameter in Nile tilapia [187] and rainbow trout [17], which negatively affected the nutrient utilization and hence, growth of the associated species. These variabilities reported here could result from the presence of several antinutritional factors (phytic acid, saponin, and tannins) in seaweeds that alter the structure of the intestine and negatively affect the digestion process [92,188]. The effects of seaweed supplementation on the histo-morphological structures of various fish species are presented in Table 4.

### 5.4. Gut Microbiota Composition

**Figure 7:**
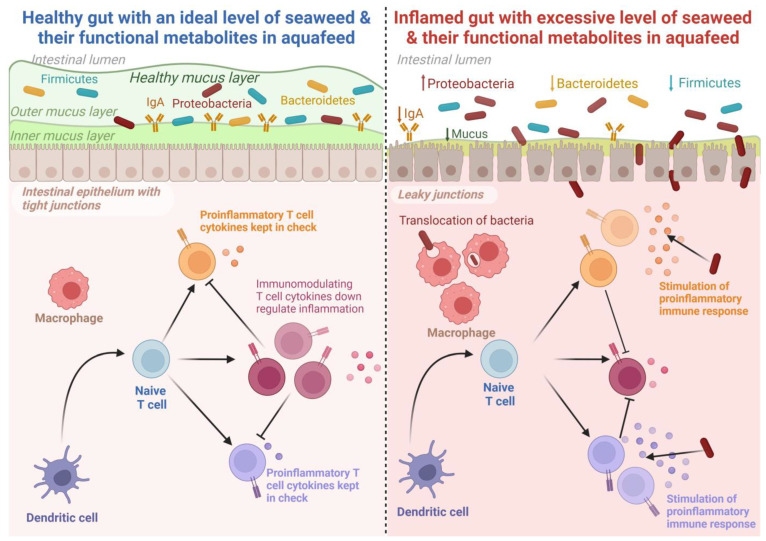
The potential effects of seaweed and seaweed-based functional metabolites in improving the gut health of fish. An optimal dietary inclusion of seaweed in aquafeed stimulates gut microbiota and improves immune response, whereas excessive inclusion is reported to suppress growth, mask immune response, and distort gut microbiota. Created with BioRender.com accessed on 25 September 2023.

The study of fish gut microbiota has attracted significant research attention in recent years [208]. Gut microbiota plays an important role in fish growth, nutrition, immunity, and resistance against pathogenic microorganisms [4,209]. The fish gut acts as an assemblage of sever al microbial communities, and their activities greatly influence different aspects of fish physiology [210]. An overview of the role of seaweed on gut microbiota composition in fish is presented in Table 4. The microbial communities in fish guts vary greatly depending on several factors such as the physiological state of the gut, trophic level and environment, and dietary ingredients [211,212]. Seaweed has been shown to be a promising feed additive that can modulate the gut microbial composition of fish [196]. The dietary inclusion of *Gracilaria gracilis* enhanced the abundance of the microbes *Sulfitobacter* and *Methylobacterium* in the gut of European seabass [197]. These microbes are capable of producing short- and medium-chain fatty acids and can lower the pH of the intestine, thus playing a crucial role in suppressing pathogenic bacteria and potentially representing a promising method of enhancing disease resistance in fish [213,214]. Similarly, dietary supplementation of fucoidan, a polysaccharide derived from brown seaweed (*Undaria pinnatifida*), is reported to elevate the intestinal digestive enzyme activities and, thereby, modulate the intestinal microbial communities in gibel carp (*Carassius auratus gibelio*) when added at a level of 30 g/kg WW [190]. Furthermore, the provision of *S. dentifolium* (3 g/kg of diet DW) extract significantly lowered the abundance of pathogenic microorganisms in the gut of Pacific white shrimp (*Litopenaeus vannamei*) [100]. On the contrary, a high inclusion (8%) of *G. gracilis* resulted in a significant reduction of gut microbial diversity. However, these negative impacts were mitigated at a lower inclusion level (4%) [197]. Tapia-paniagua et al. [198] reported that a relatively low (<3%) dietary administration of *Ulva ohnoi* significantly enhanced the diversity of whole gut microbes in Senegalese sole (*Solea senegalensis*), while 5% *U. ohnoi* did not exhibit a significant influence on gut microbial diversity [196]. These variable results could be attributed to the specific adaptative response of different microbial communities across a range of feeding schedules, such as time and duration of feeding and feeding frequencies. Nevertheless, dietary *U. ohnoi* reduced the abundance of the genus *Escherichia* [198] in *S. senegalensis*, which could be attributed to the antibacterial properties of *Ulva* spp. against *Escherichia coli* [215,216]. Further, Xinxu et al. [206] reported that dietary *Ulva australis* (formerly *Ulva pertusa*) (Chlorophyta) enhanced the abundance of several bacterial species of the Firmicutes group, including *Ruminococcus*, *Clostridium*, and Lachnospiraceae in white-spotted rabbitfish (*S. canaliculatus*) that actively participate in the degradation of non-starch polysaccharides in the host gut [217,218]. These results indicate that seaweed inclusion in aquafeed can be beneficial up to a certain extent, while the excessive inclusion of seaweed in aquafeed can lead to negative effects, including reductions in the growth of beneficial gut bacteria, leading to poor digestion and nutrient absorption, which may weaken fish immune systems and subsequently increase susceptibility to disease. The potential impacts of dietary seaweed inclusion or their extracts on the intestinal health of fish are depicted in Figure 7.

## 6. Potential Limitations and Future Perspectives

While seaweed offers many advantages as a potential ingredient for aquafeed, there are some potential disadvantages that need to be considered. A summary through SWOT (strength–weakness–opportunity–threat) analysis of seaweed utilization as an aquafeed ingredient and source of bioactive metabolites is presented in Table 5. These will be addressed below while also discussing potential mitigation strategies and future perspectives.

Significant industry expansion required globally: Commercial seaweed farming takes place in over 35 countries worldwide, but the bulk (98.9%) is concentrated in China (60%) and Southeast Asia, notably Indonesia (21%), the Philippines (9%), and Malaysia (1%) [219]. Small subsistence farms (1 ha) abound throughout Southeast Asia, and their expansion is largely regulated by both access to usable habitats and proximity to markets. Therefore, seaweed production must continue to expand into other regions as the demand for seaweed and seaweed derivatives grows. Clearly, the expansion of the seaweed aquaculture industry into other parts of the world will be subject to both logistical and regulatory constraints, and this will ultimately determine the viability of continued industry expansion. As such, regionally specific viability assessments for seaweed aquaculture will be continually required to meet the growing needs within both the human and animal feed sectors.

Nutrient variability: Seaweeds are known to have variable nutrient compositions depending on factors such as species, growing conditions, and harvesting season [220]. This variability can make it challenging to formulate consistent and balanced diets for farmed aquatic organisms. To combat this, additional compositional analysis and feed formulation adjustments are required to ensure the nutritional requirements of the target species are met consistently.

Limited availability: Seaweeds have specific growth seasons, and their availability can be influenced by factors such as temperature, light, and nutrient availability [221]. Seasonal fluctuations in seaweed production can affect the stability and continuity of the aquafeed supply chain, potentially leading to feed shortages or increased costs during certain times of the year [222]. Therefore, understanding seasonal patterns, diversification of seaweed farming techniques (i.e., floating or suspended cultivation systems), and post-harvest preservation could be maintained to create a more stable and sustainable seaweed production system.

Lower inclusion level: The benefits of dietary seaweed inclusion are generally best realized when included in aquafeed at levels of around 5%. Beyond this level, a host of negative impacts on growth and feed utilization in fish have been reported, in part, due to palatability issues. Therefore, more research is needed to determine the most appropriate seaweed species and their optimal inclusion levels in aquafeed for fish and also whether their inclusion is most appropriate when utilized as a minor ingredient or functional additive. Furthermore, fermentation or enzymatic processing should be explored to improve the functionality of seaweed inclusion in aquafeed for farmed fish.

Processing challenges: The processing of seaweed into suitable raw materials for inclusion into aquafeed can present certain challenges. Namely, seaweeds have a high moisture content, which needs to be reduced to enhance shelf life and prevent spoilage. Processing methods, such as drying or grinding, inevitably require energy and infrastructure, which can add to the overall cost of production.

Palatability and acceptance: Seaweeds have distinct flavors, textures, and high ash contents that may not be universally accepted by all species of farmed fish [223]. Some fish species may show lower feed intake or reduced growth rates when seaweed-based diets are used. For example, excessive ash can lead to reduced digestibility of feed since most aquaculture species have their specific nutritional requirements. The application of fermentation or enzymatic processing may increase seaweed palatability. However, encouraging feed acceptance and addressing palatability issues might require additional research and formulation adjustments.

Heavy metals contamination: Hazardous levels of heavy metals (such as cadmium, lead, mercury, and arsenic) and iodine in some seaweed species may pose a barrier to their use in aquafeed [224]. Furthermore, the presence of antinutritional components, radioactive isotopes, ammonium, dioxins, and pesticides is an important consideration. Previous research has also shown that wild seaweed may include traces of plastic particles, raising concerns about the toxicity of seaweed in aquafeed. More feeding and clinical experiments are needed to better understand the bioavailability of pollutants and other limiting variables that may prevent its utilization in aquafeed.

## 7. Conclusions

The nutritional and functional properties of seaweed attest to their potential to be incorporated into aquafeed to safeguard fish growth and health as the global demand for fish and seafood products rapidly increases. An increasing body of research demonstrates that seaweed and seaweed-derived functional metabolite supplementation has a major positive effect on growth, physiological stress resilience, and immune response in fish. However, additional research into the challenges of utilizing seaweed is warranted, specifically on the effect of seaweed supplementation on nutrient digestibility, the potential of long-term effects on fish health, and the possible interactive effects between dietary seaweed supplementation with high dietary inclusion levels of terrestrial plant proteins and carbohydrates. Furthermore, the potential effects of seaweed and its immunomodulatory compounds should be further explored in fish to elucidate their underlying physiological mechanisms. Such research is deemed necessary to fully understand the existing commercial potential of seaweed in aquaculture and in order to advance this emerging field of research. Finally, the standardization of study conditions and subsequent reporting of key metrics relating to fish health and performance will aid in the elucidation of optimal inclusion levels for seaweed and seaweed-derived bioactives in aquafeed for a multitude of species. Ultimately, this will support the future widespread use of seaweed and seaweed-derived bioactives in aquaculture production.

## Figures and Tables

**Table 1:** Bioactive compounds in various seaweeds.

Seaweed Species	Tested Solvent	Bioactive Compounds	Concentration	Reference
Red seaweed (Rhodophyta)				
*Porphyra umbilicalis*	Methanol	TPC	5.53 g GAE/100 g DW	[63]
*P. umbilicalis*	90% acetone	Carotenoid Chl *a*	1.88 µg/g WW	[64]
*Jania rubens*			38.41 µg/mL WW	
*Hypnea musciformis*	Ethyl acetate	TPC	205.5 mg GAE/g DW	[65]
*Gracilaria edulis*	Aqueous fraction	TPC	1704.69 µg GAE/g DW	[66]
		TFC	786.95 µg QE/g DW	
		Alkaloids	522.34 µg PEG/g DW	
*Gracilaria tenuistipitata*	Methanol	TPC	68.20 mg GAE/g DW	[67]
		TFC	36.17 mg QE/g DW	
*Acanthophora spicifera*	Ethyl acetate	TPC	40.583 µg/mg DW	[68]
Green seaweed (Chlorophyta)				
*Ulva lactuca*	Water	TPC	4.60 mg/g DW	[69]
		TChl	21.27 mg/g DW	
		Carotenoids	12.73 mg/g DW	
*Ulva lactuca*	Methanol	Fucoxanthin	7.53 mg/g DW	[70]
*Ulva intestinalis*	Dichloromethane	TPC	197.6 mg GAE/g extract	[71]
*Caulerpa lentillifera*	Methanol	TPC	42.85 mg PGE/g DW	[72]
Brown seaweed (Phaeophyceae)				
*Undaria pinnatifida*	Methanol	TPC	4.46 g GAE/100 g DW	[63]
*Saccharina japonica*	Distilled water	Carotenoids	2.391 mg/g DW	[73]
*Halopteris scoparia*	Methanol	TPC	328.7 mg GAE/100 g DW	[55]
	Ethanol		123.2 mg GAE/100 g DW	
	Water		328.7 mg GAE/100 g DW	
*Sargassum* sp.	Acetone	TPC	14.6 mg GAE/g DW	[74]
		TFC	0.67 mg QE/g DW	
*Ecklonia radiata*	Methanol	TPC	12.19 mg GAE/g DW	[74]
		TFC	11.15 mg QE/g DW	
*Himanthalia elongata*	Methanol	Polyphenol	23.47 g GAE/100 g DW	[63]
*Ascophyllum nodosum*	Water	Fucoxanthin	172–660 mg/kg DW	[75]
*Himanthalia elongate*	60% methanol	TPC	286.0 mg GAE/g DW	[76]
		TFC	109.8 mg QE/g DW	

Note: WW—wet weight; DW—dry weight; TPC—total phenolic content; TFC—total flavonoid content; PGE—phloroglucinol equivalents; GAE—gallic acid equivalence; QE—quercetin; PEG—polyethylene glycol; Chl *a*—chlorophyll a; TChl—total chlorophyll.

**Table 2:** Major seaweeds used in aquafeed as the sources of nutrients and bioactive compounds for fish.

Seaweed Group	Genus	Species
Red seaweed (Rhodophyta)	*Chondrus*	*C. crispus*
	*Kappaphycopsis* (formerly *Eucheuma*)	*E. cottonii*
		*E. denticulatum*
	*Palmaria*	*Palmaria palmata*
	*Gracilaria*	*G. edulis* (formerly *G. lichenoides*)
		*G. heteroclada*
		*G. lichenoides*
		*G. cornea*
		*G. crassa*
		*G. gracilis*
		*G. persica*
		*G. vermiculophylla*
		*G. pulvinata*
	*Porphyra*	*P. purpurea*
		*Pyropia yezoensis* (*Porphyra yezoensis*)
	*Gracilariopsis*	*G. persica*
		*G. lemaneiformis*
Brown seaweed (Phaeophyceae)	*Sargassum*	*S. fusiforme*
		*S. portieranum*
		*S. aquifolium*
		*S. horneri*
		*S. boveanum*
		*S. angustifolium*
		*S. dentifolium*
		*S. fulvellum*
	*Saccharina* (formerly *Laminaria*)	*S. japonica*
		*S. latissimi*
	*Ascophyllum*	*A. nodosum*
	*Saccharina*	*S. japonica*
		*S. latissimi*
	*Undaria*	*U. pinnatifida*
	*Cladosiphon*	*C. okamuranus*
	*Padina*	*P. gymnospora*
		*P. pavonica*
	*Macrocystis*	*M. pyrifera*
Green seaweed (Chlorophyta)	*Ulva*	*U. lactuca*
		*U. rigida*
		*U. fascita*
		*U. reticulata*
		*U. autralis* (formerly *U. pertusa*)
		*U. prolifera*
		*U. intestinalis*
		*U. ohnoi*
	*Capsosiphon*	*C. fulvescens*
	*Codium*	*C. fragile*
	*Monostroma*	*M. nitidum*
	*Caulerpa*	*C. lentillifera*

**Table 4:** The effects of seaweed and seaweed-based functional metabolites on gut histo-morphometry and gut microbiota composition in farmed fish (studied parameters were compared to control—0% FM diet).

Seaweed and Derivatives	Fish Species	Applied Levels	Effective Level	Response	Reference
*Ulva* sp. (Chlorophyta), *Gracilaria gracilis* (Rhodophyta)	*D. labrax*	2 and 4%	-	SW-blend-supplemented diet enhanced anterior intestinal absorption area by up to 45%	[189]
Fucoidan from *Undaria pinnatifida* (Phaeophyceae)	*Carassius auratus gibelio*	0.1, 1.0 and 3.0%	3.0%	Increased intestinal digestive enzyme activity, thereby enhancing intestinal microbial communities at a level of 3% dietary supplementation	[190]
Fucoidan from *Undaria pinnatifida* (Phaeophyceae)	*Salmo salar*	1 and 3%	-	Fucoidan positively improved intestinal integrity and immune response	[191]
Fucoidan from *Saccharina japonica* (Phaeophyceae)	*O. niloticus*	0.1, 0.2, 0.4, and 0.8%	-	Fucoidan in fish diets improved intestinal health and antioxidant status	[192]
*Sargassum dentifolium* (Phaeophyceae)	*O. mossambicus × O. niloticus*	1, 2, and 3%	-	No abnormal or histological changes were detected due to the dietary SW supplementation	[143]
*Sargassum ilicifolium* (Phaeophyceae)	*L. calcarfer*	3, 6, and 9%	6%	No significant difference observed between enterocyte length, villi width, and muscle thickness in intestinal tissue between different treatments and the control group	[180]
*Spirulina platensis*	*L. calcarifer*	10, 20, and 40%	20%	Decreased intestinal fold and microvilli height were observed in fish fed 40% of *Spirulina* sp. in the diet	[193]
*Pelvetia canaliculata* (Phaeophyceae)	*S. aurata*	1 and 10%	10%	10% SW supplementation led to greater thickness of the muscle layers and longer villi length	[194]
*Gracilaria gracilis* (Rhodophyta)	*D. labrax*	0.35, 2.5, and 5%	2.5%	2.5% SW inclusion boosted the intestinal acid goblet cells	[183]
*G. gracilis* (Rhodophyta) and the microalga *Nannochloropsis oceanica* (Eustigmatophyceae)	*D. labrax*	8%	-	All fish had well-preserved gut morphology; however, significant enhancement of goblet cells was observed in *Nannochloropsis*-based diet compared to *Gracilaria*-based feed	[184]
*Ulva ohnoi* (Chlorophyta)	*S. senegalensis*	5%	-	SW significantly reduced damage to intestinal mucosa and enhanced the mucosal absorptive surface area	[91]
*Laminaria* sp.	*S. salar*	3, 6, and 10%	-	Higher gut and intestinal weights and lengths were observed due to dietary SW provision. Lager surface area exhibited	[82]
*Ulva lactuca* (Chlorophyta), *Chondrus crispus* (Rhodophyta)	*S. aurata*	2.5 and 5%	-	Dietary SW had no significant on distal intestine histomorphology	[195]
*Gracilaria pygmaea* (Rhodophyta)	*O. mykiss*	3, 6, 9, and 12%	9 and 12%	Normal histomorphology of anterior intestine and pyloric caeca was detected. Villi decreased due to 90 and 120 g/kg provision of SW	[84]
*Ulva rigida* (Chlorophyta), *Undaria pinnatifida* (Phaeophyceae)	*S. senegalensis*	10%	-	Dietary *Undaria* significantly lowered the width of intestine villi	[101]
*Taonia atomaria* (Phaeophyceae)	*O. niloticus*	5, 10, and 15%	-	No histopathological alterations were observed due to dietary SW provision	[138]
*Asparagopsis taxiformis* (Rhodophyta)	*S. salar*	1.8, 2.6, and 3%	-	Increased bacteria diversity found in the hindgut	[23]
*Gracilaria cornea* (Rhodophyta), *Ulva rigida* (Chlorophyta)	*S. aurata*	5, 15, and 25%	-	SW inclusion did not reveal any negative effects on gut structure	[93]
*Gracilaria vermiculophylla, Porphyra dioica* (Rhodophyta), and *Ulva* spp. (Chlorophyta)	*O. niloticus*	10%	-	Exhibited a significant reduction in villi length in *Gracilaria*- and *Porphyra*-based diets, while no significant reduction was observed in case of *Ulva* spp.	[187]
*Ulva ohnoi* (Chlorophyta)	*S. senegalensis*	5%	-	SW supplementation significantly enhanced the abundance of *Vibrio* while decreasing *Stenotrophomonas* abundance	[196]
*Gracilaria gracilis* (Rhodophyta)	*D. labrax*	8%	*-*	*G. gracilis* supplementation promoted the growth of *Sulfitobacter* and *Methylobacterium*	[197]
*Ulva ohnoi* (Chlorophyta)	*S. senegalensis*	5%	*-*	*Pseudomonas* and *Mycopasmataceae* were abundant in anterior and posterior GI tract, respectively	[198]
*Gracilaria gracilis* (Rhodophyta)	*D. labrax*	2.5 and 5%	-	Gut microbiome diversity was not altered by SW supplementation. Abundance of Proteobacteria was reduced	[199]
*Ulva rigida* (Chlorophyta)	*S. aurata*	25%	-	SW supplementation significantly modified intestinal microbiota	[200]
*Sargassum angustifolium* (Phaeophyceae), *Gracilaria pulvinata* (Rhodophyta)	*O. mykiss*	0.025 and 0.05%	-	Supplementation of SW extracts did not affect total bacterial level; however, the abundance of *Lactobacillus* increased	[201]
*Gracilaria* sp. (Rhodophyta)	*S. aurata*	2.5 and 5%	5%	Abundance of Firmicutes phyla and *Clostridium* genera were enhanced with 5% SW	[202]
*Ulva rigida* (Chlorophyta), *Ascophyllum nodosum* (Rhodophyta)	*Gadus morhua*	10%	*-*	*U. rigida* did not significantly influence the microbial composition of hindgut, while *A. nodosum* altered the scenario	[203]
Mixture of red, brown, and green SW	*Siganus fuscescens*	-	-	Increased abundance of *Firmicutes* and *Proteobacteria* while decreasing *Bacteroides*	[204]
*Laminaria* sp. (Alginates)	*S. salar*	0.5 and 2.5%	0.5%	Facilitated the abundance of several Proteobacteria such as *Photobacterium phosphoreum*, *Aquabacterium parvum*, and *Achromobacter insolitus*	[205]
*Ulva australis* (formerly *Ulva pertusa*) (Chlorophyta)	*S. canaliculatus*	10%	-	SW in the diets enhanced the diversity of Firmicutes, Bacteroidetes, and Proteobacteria	[206]
*Gracilaria cornea* (Rhodophyta), *Ulva rigida* (Chlorophyta)	*S. aurata*	5, 15, and 25%	15%	Biodiversity of microbial community was significantly reduced with highest inclusion of *U*. *rigida.* Various *Lactobacillus* sp. were significantly stimulated, while *Vibrio* sp. was reduced	[207]

Note: SW—seaweed; GI—gastrointestinal tract; - — not identified.

**Table 5:** SWOT analysis of seaweed utilization as aquafeed ingredient and source of feed additives (seaweed-based bioactive metabolites) in aquaculture production.

Strengths	Weaknesses
Low production cost Natural Sustainable Non-toxic Non-drug resistance Low impact on the environment Health-promoting molecules	High extraction cost High nutrient variability Heavy metal contaminations Limited availability across the globe Rigid cell wall leading to lower digestibility
**Threats**	**Opportunities**
Irregular supply Inadequate safety evaluation Seawater and seaweed quality Overexploitation Introduction of alien species Climate change	Value addition Demand for high-quality seafood Expanding aquaculture industry Potential to use for drug development Diverse species and scope of genetic modification

## Data Availability

Data are contained within the article.

## References

[1] Garlock T., Asche F., Anderson J., Ceballos-concha A., Love D.C., Osmundsen T.C., Beatriz R., Pincinato M. (2022). Aquaculture: The missing contributor in the food security agenda. Glob. Food Secur..

[2] Barasa J.E., Mukhongo P.N., Ngetich C.C. (2022). Perspectives on salmon aquaculture: Current status, challenges and genetic improvement for future growth. Salmon Aquaculture.

[3] Afewerki S., Asche F., Misund B., Thorvaldsen T., Tveteras R. (2023). Innovation in the Norwegian aquaculture industry. Rev. Aquac..

[4] Wan A.H.L., Davies S.J., Soler-vila A., Fitzgerald R., Johnson M.P. (2019). Macroalgae as a sustainable aquafeed ingredient. Rev. Aquac..

[5] Akbary P., Aminikhoei Z. (2018). Effect of water-soluble polysaccharide extract from the green alga Ulva rigida on growth performance, antioxidant enzyme activity, and immune stimulation of grey mullet *Mugil cephalus*. J. Appl. Phycol..

[6] Thepot V., Campbell A.H., Rimmer M.A., Paul N.A. (2021). Meta-analysis of the use of seaweeds and their extracts as immunostimulants for fish: A systematic review. Rev. Aquac..

[7] Dawczynski C., Schubert R., Jahreis G. (2007). Amino acids, fatty acids, and dietary fibre in edible seaweed products. Food Chem..

[8] Saleh H.H. (2020). Review on using of macroalgae (seaweeds) in fish nutrition. J. Zool. Res..

[9] Øverland M., Mydland L.T., Skrede A. (2019). Marine macroalgae as sources of protein and bioactive compounds in feed for monogastric animals. J. Sci. Food Agric..

[10] Martínez-Antequera F.P., Martos-Sitcha J.A., Reyna J.M., Moyano F.J. (2021). Evaluation of the Inclusion of the Green Seaweed Ulva ohnoi as an Ingredient in Feeds for Gilthead Sea Bream (*Sparus aurata*) and European Sea Bass (*Dicentrarchus labrax*). Animals.

[11] Satoh K.-I., Nakagawa H., Kasahara S. (1987). Effect of Ulva meal supplementation on disease resistance of red sea bream. Bull. Jpn. Soc. Sci. Fish..

[12] Soler-vila A., Coughlan S., Guiry M.D. (2009). The red alga *Porphyra dioica* as a fish-feed ingredient for rainbow trout (*Oncorhynchus mykiss*): Effects on growth, feed efficiency, and carcass composition. J. Appl. Phycol..

[13] An B.N.T., Anh N.T.N. (2020). Co-culture of Nile tilapia (*Oreochromis niloticus*) and red seaweed (*Gracilaria tenuistipitata*) under different feeding rates: Effects on water quality, fish growth and feed efficiency. J. Appl. Phycol..

[14] Wassef E.A., El-Sayed A., Sakr E.M. (2013). *Pterocladia* (Rhodophyta) and *Ulva* (Chlorophyta) as feed supplements for European seabass, *Dicentrarchus labrax* L., fry. J. Appl. Phycol..

[15] Azaza B.M.S., Mensi F., Ksouri J., Dhraief M.N., Brini B., Abdelmouleh A., Kraı M.M. (2008). Growth of Nile tilapia (*Oreochromis niloticus* L.) fed with diets containing graded levels of green algae *Ulva* meal (*Ulva rigida*) reared in geothermal waters of southern Tunisia. J. Appl. Ichthyol..

[16] Rocha C.P., Pacheco D., Cotas J., Marques J.C., Pereira L., Gonçalves A.M.M. (2021). Seaweeds as valuable sources of essential fatty acids for human nutrition. Int. J. Environ. Res. Public Health.

[17] De Carvalho C.C.C.R., Caramujo M.J. (2018). The Various Roles of Fatty Acids. Molecules.

[18] Peñalver R., Lorenzo J.M., Ros G., Amarowicz R., Pateiro M., Nieto G. (2020). Seaweeds as a functional ingredient for a healthy diet. Mar. Drugs.

[19] Gora A.H., Sahu N.P., Sahoo S., Rehman S., Dar S.A., Ahmad I., Agarwal D. (2018). Effect of dietary *Sargassum wightii* and its fucoidan-rich extract on growth, immunity, disease resistance and antimicrobial peptide gene expression in *Labeo rohita*. Int. Aquat. Res..

[20] Safavi S.V., Kenari A.A., Tabarsa M., Esmaeili M. (2019). Effect of sulfated polysaccharides extracted from marine macroalgae (*Ulva intestinalis* and *Gracilariopsis persica*) on growth performance, fatty acid profile, and immune response of rainbow trout (*Oncorhynchus mykiss*). J. Appl. Phycol..

[21] Thanigaivel S., Chandrasekaran N., Mukherjee A., Thomas J. (2019). Protective efficacy of microencapsulated seaweed extracts for preventing *Aeromonas* infections in *Oreochromis mossambicus*. Comp. Biochem. Physiol..

[22] Vazirzadeh A., Marhamati A., Rabiee R., Faggio C. (2020). Immunomodulation, antioxidant enhancement and immune genes up-regulation in rainbow trout (*Oncorhynchus mykiss*) fed on seaweeds included diets. Fish Shellfish Immunol..

[23] Thepot V., Campbell A.H., Rimmer M.A., Jelocnik M., Johnston C. (2022). Dietary inclusion of the red seaweed *Asparagopsis taxiformis* boosts production, stimulates immune response and modulates gut microbiota in Atlantic salmon, *Salmo salar*. Aquaculture.

[24] Siddik M.A.B., Vatsos I.N., Rahman M.A., Pham H.D. (2022). Selenium-Enriched Spirulina (SeE-SP) Enhance Antioxidant Response, Immunity, and Disease Resistance in Juvenile Asian Seabass, *Lates calcarifer*. Antioxidants.

[25] Murty U.S., Banerjee A.K. (2012). Seaweeds: The wealth of oceans. Handbook of Marine Macroalgae: Biotechnology and Applied Phycology.

[26] Din N.A.S., Mohd Alayudin S., Sofian-Seng N.S., Rahman H.A., Mohd Razali N.S., Lim S.J., Wan Mustapha W.A. (2022). Brown algae as functional food source of fucoxanthin: A review. Foods.

[27] Misurcova L. (2011). Isolation and chemical properties of molecules derived from seaweeds chemical composition of seaweeds. Handbook of Marine Macroalgae.

[28] Salehi B., Sharifi-rad J., Seca A.M.L., Pinto D.C.G.A. (2019). Current trends on seaweeds: Looking at chemical composition, phytopharmacology, and cosmetic applications. Molecules.

[29] Li Y., Zheng Y., Zhang Y., Yang Y., Wang P., Imre B., Wong A.C.Y., Hsieh Y.S.Y., Wang D. (2021). Brown Algae Carbohydrates: Structures, Pharmaceutical Properties, and Research Challenges. Mar. Drugs.

[30] D’Armas H.D., Jaramillo C., Armas M.D., Echavarría A., Valverde P. (2019). Proximate composition of several macroalgae from the coast of Salinas Bay, Ecuador. Rev. Biol. Trop..

[31] Schmid M., Kraft L.G.K., Van Der Loos L., Kraft G.T., Virtue P., Nichols P.D., Hurd C.L. (2018). Southern Australian seaweeds: A promising resource for omega-3 fatty acids. Food Chem..

[32] Airanthi M.K.W., Sasaki N., Iwasaki S., Baba N., Abe M., Hosokawa M., Miyashita K. (2011). Effect of brown seaweed lipids on fatty acid composition and lipid hydroperoxide levels of mouse liver. J. Agric. Food Chem..

[33] Hamed I., Özogul F., Özogul Y., Regenstein J.M. (2015). Marine bioactive compounds and their health benefits: A review. Compr. Rev. Food Sci. Food Saf..

[34] Holdt S.L., Kraan S. (2011). Bioactive compounds in seaweed: Functional food applications and legislation. J. Appl. Phycol..

[35] Rawiwan P., Peng Y., Paramayuda I., Quek S. (2022). Red seaweed: A promising alternative protein source for global food sustainability. Trends Food Sci. Technol..

[36] Cian R.E., Drago S.R., de Medina F.S., Martínez-Augustin O. (2015). Proteins and carbohydrates from red seaweeds: Evidence for beneficial effects on gut function and microbiota. Mar. Drugs.

[37] Renaud S.M., Luong-van J.T. (2006). Seasonal variation in the chemical composition of tropical Australian marine macroalgae. J. Appl. Phycol..

[38] Pereira L. (2011). A review of the nutrient composition of selected edible seaweeds. Seaweed.

[39] Sanchez-Machado D.I., Lopez-Cervantes J., López-Hernández J., Paseiro-Losada P. (2004). Fatty acids, total lipid, protein and ash contents of processed edible seaweeds. Food Chem..

[40] Gaspar R., Fonseca R., Pereira L. (2020). Illustrated Guide to the Macroalgae of Buarcos Bay, Figueira da Foz, Portugal.

[41] Wong K.H., Cheung P.C.K. (2000). Nutritional evaluation of some subtropical red and green seaweeds: Part I—Proximate composition, amino acid profiles and some physico-chemical properties. Food Chem..

[42] Benjama O., Masniyom P. (2011). Nutritional composition and physicochemical properties of two green seaweeds (*Ulva pertusa* and *U. intestinalis*) from the Pattani bay in southern Thailand. Songklanakarin J. Sci. Technol..

[43] Rajapakse N., Kim S.K. (2011). Nutritional and digestive health benefits of seaweed. Advances in Food and Nutrition.

[44] Corino C., Modina S.C., Di Giancamillo A., Chiapparini S. (2019). Seaweeds in pig nutrition. Animals.

[45] Garcı M.N., Pereira A.C., Leets I., Quiroga M.F. (2018). High iron content and bioavailability in humans from four species of marine algae. J. Nutr..

[46] Kumar C.S., Ganesan P., Pv S., Bhaskar N. (2008). Seaweeds as a source of nutritionally beneficial compounds—A review. J. Food Sci. Technol..

[47] Charoensiddhi S., Conlon M.A., Vuaran M.S., Franco C.M.M., Zhang W. (2017). Polysaccharide and phlorotannin-enriched extracts of the brown seaweed *Ecklonia radiata* influence human gut microbiota and fermentation in vitro. J. Appl. Phycol..

[48] Sari-chmayssem N., Taha S., Mawlawi H., Guégan J., Jefti J., Benvegnu T. (2018). Extracted ulvans from green algae *Ulva linza* of lebanese origin and amphiphilic derivatives: Evaluation of their physico-chemical and rheological properties. J. Appl. Phycol..

[49] Murata M., Nakazoe J. (2001). Production and use of marine algae in Japan. Jpn. Agric. Res. Q..

[50] Marinho-Soriano E., Fonseca P., Carneiro M., Moreira W. (2006). Seasonal variation in the chemical composition of two tropical seaweeds. Bioresour. Technol..

[51] Craigie J.S. (2011). Seaweed extract stimuli in plant science and agriculture. J. Appl. Phycol..

[52] Devill C., Damas J., Forget P., Dandrifosse G., Peulen O. (2004). Laminarin in the dietary fibre concept. J. Sci. Food Agric..

[53] Cotas J., Leandro A., Monteiro P., Pacheco D., Figueirinha A., Gonçalves A.M.M., da Silva G.J., Pereira L. (2020). Seaweed phenolics: From extraction to applications. Mar. Drugs.

[54] Gunathilake T., Akanbi T.O., Suleria H.A.R., Nalder T.D., Francis D.S., Barrow C.J. (2022). Seaweed phenolics as natural antioxidants, aquafeed additives, veterinary treatments and cross-linkers for microencapsulation. Mar. Drugs.

[55] López A., Rico M., Rivero A., de Tangil M.S. (2011). The effects of solvents on the phenolic contents and antioxidant activity of *Stypocaulon scoparium* algae extracts. Food Chem..

[56] Yumiko Y., Ya-Pei H., Takeshi S. (2003). Distribution of flavinoids and related compounds from seaweeds in Japan. J. Tokyo Univ. Fish..

[57] Onofrejová L., Vašíčková J., Klejdus B., Stratil P., Mišurcová L., Kráčmar S., Kopecký J., Vacek J. (2010). Bioactive phenols in algae: The application of pressurized-liquid and solid-phase extraction techniques. J. Pharm. Biomed. Anal..

[58] Catarino M.D., Silva A.M.S., Cardoso S.M. (2017). Fucaceae: A source of bioactive phlorotannins. Int. J. Mol. Sci..

[59] El-beltagi H.S., Mohamed A.A., Mohamed H.I., Ramadan K.M.A. (2022). Phytochemical and potential properties of seaweeds and their recent applications: A review. Mar. Drugs.

[60] Jensen A. (1966). Carotenoids of Norwegian Brown Seaweeds and of Seaweed Meals.

[61] Balasubramaniam V., Chelyn L.J., Vimala S., Fairulnizal M.N.M., Brownlee I.A., Amin I. (2020). Carotenoid composition and antioxidant potential of *Eucheuma denticulatum*, *Sargassum polycystum* and *Caulerpa lentillifera*. Heliyon.

[62] Osório C., Machado S., Peixoto J., Bessada S., Pimentel F.B., Alves R.C., Oliveira M.B.P.P. (2020). Pigments content (Chlorophylls, Fucoxanthin and Phycobiliproteins) of different commercial dried algae. Separations.

[63] Cofrades S., López-Lopez I., Bravo L., Ruiz-Capillas C., Bastida S. (2010). Nutritional and antioxidant properties of different brown and red spanish edible seaweeds. Food Sci. Technol. Int..

[64] Freitas M.V., In L.G., Martins M., Pereira L., Mouga T. (2022). Primary composition and pigments of 11 red seaweed species from the center of Portugal. J. Mar. Sci. Eng..

[65] Chakraborty K., Joseph D. (2015). Antioxidant activities and phenolic contents of three red seaweeds (Division: Rhodophyta) harvested from the gulf of mannar of Peninsular India. J. Food Sci. Technol..

[66] Gunathilaka T.L., Samarakoon K.W., Ranasinghe P., Peiris L.D.C. (2019). In-Vitro Antioxidant, Hypoglycemic Activity, and Identification of Bioactive Compounds in Phenol-Rich Extract from the Marine Red Algae Gracilaria edulis (Gmelin) Silva. Molecules.

[67] Sobuj M.K.A., Islam M.A., Islam M.S., Islam M.M., Mahmud Y., Rafiquzzaman S.M. (2021). Effect of Solvents on Bioactive Compounds and Antioxidant Activity of *Padina tetrastromatica* and *Gracilaria tenuistipitata* Seaweeds Collected from Bangladesh. Sci. Rep..

[68] Zakaria N.A., Ibrahim D., Sulaiman S.F., Supardy N.A., Biotechnology I., Sains U., Pinang P., Pinang P. (2011). Assessment of antioxidant activity, total phenolic content and in-vitro toxicity of Malaysian red seaweed, *Acanthophora spicifera*. J. Chem. Pharm. Res..

[69] El-Baky H.A., El Baz F.K., Baroty G.S.E. (2008). Evaluation of marine alga *Ulva lactuca* L. as a source of natural preservative ingredient. Am.-Eurasian J. Agric. Environ. Sci..

[70] Roh M., Uddin S., Chun B. (2008). Extraction of fucoxanthin and polyphenol from *Undaria pinnatifida* using supercritical carbon dioxide with co-solvent. Biotechnol. Bioprocess Eng..

[71] Srikong W., Bovornreungroj N., Mittraparparthorn P. (2017). Antibacterial and antioxidant activities of differential solvent extractions from the green seaweed *Ulva intestinalis*. ScienceAsia.

[72] Rattaya S., Benjakul S. (2015). Extraction, antioxidative, and antimicrobial activities of brown seaweed extracts, *Turbinaria ornata* and *Sargassum polycystum*, grown in Thailand. Int. Aquat. Res..

[73] Saravana P., Cho Y., Park Y., Woo H., Chun B. (2016). Structural, antioxidant, and emulsifying activities of fucoidan from *Saccharina japonica* using pressurized liquid extraction. Carbohydr. Polym..

[74] Subbiah V., Ebrahimi F., Agar O.T., Dunshea F.R., Barrow C.J., Suleria H.A.R. (2023). Comparative Study on the Effect of Phenolics and Their Antioxidant Potential of Freeze-Dried Australian Beach-Cast Seaweed Species upon Different Extraction Methodologies. Pharmaceuticals.

[75] Apostolidis E., Lee C.M. (2010). In vitro potential of *Ascophyllum nodosum* phenolic antioxidant mediated α-glucosidase and α-amylase inhibition. J. Food Sci..

[76] Rajauria G., Jaiswal A.K., Abu-gannam N., Gupta S. (2013). Antimicrobial, antioxidant and free radical-scavenging capacity of brown seaweed *Himanthalia elongata* from western coast of Ireland. J. Food Biochem..

[77] FAO (2021). Top 10 Species Groups in Global Aquaculture 2019. World Aquaculture Performance Indicators (WAPI) Factsheet. www.fao.org/3/cb5186en/cb5186en.pdf.

[78] Valente L.M.P., Gouveia A., Rema P., Matos J. (2006). Evaluation of three seaweeds *Gracilaria bursapastoris*, *Ulva rigida* and *Gracilaria cornea* as dietary ingredients in European seabass (*Dicentrarchus labrax*) juveniles. Aquaculture.

[79] Tolentino-Pablico G., Bailly N., Froese R., Elloran C. (2008). Seaweeds preferred by herbivorous fishes. J. Appl. Phycol..

[80] Norambuena F., Hermon K., Skrzypczyk V., Emery J.A., Sharon Y., Beard A., Turchini G.M. (2015). Algae in fish feed: Performances and fatty acid metabolism in juvenile Atlantic salmon. PLoS ONE.

[81] Peixoto M.J., Svendsen J.C., Malte H., Pereira L.F., Carvalho P., Pereira R., Gonçalves J.F.M., Ozório R.O.A. (2016). Diets supplemented with seaweed affect metabolic rate, innate immune, and antioxidant responses, but not individual growth rate in European seabass (*Dicentrarchus labrax*). J. Appl. Phycol..

[82] Kamunde C., Sappal R., Melegy T.M. (2019). Brown seaweed (AquaArom) supplementation increases food intake and improves growth, antioxidant status and resistance to temperature stress in Atlantic salmon, *Salmo salar*. PLoS ONE.

[83] Sony N.M., Ishikawa M., Hossain S., Koshio S., Yokoyama S. (2019). The effect of dietary fucoidan on growth, immune functions, blood characteristics and oxidative stress resistance of juvenile red sea bream, *Pagrus major*. Fish Physiol. Biochem..

[84] Sotoudeh E., Mardani F. (2018). Antioxidant- related parameters, digestive enzyme activity and intestinal morphology in rainbow trout (*Oncorhynchus mykiss*) fry fed graded levels of red seaweed, *Gracilaria pygmaea*. Aquac. Nutr..

[85] Gupta S., Abu-ghannam N. (2011). Bioactive potential and possible health effects of edible brown seaweeds. Trends Food Sci. Technol..

[86] Lordan S., Ross R.P., Stanton C. (2011). Marine bioactives as functional food ingredients: Potential to reduce the incidence of chronic diseases. Mar. Drugs.

[87] Yone Y., Furuichi M., Urano K. (1985). Effects of wakame *Undaria pinnatifida* and *Ascophyllum nodosum* on absorption of dietary nutrients, and blood sugar and plasma free amino-N levels of red sea bream. Bull. Jpn. Soc. Sci. Fish..

[88] Hashim R., Azam N., Saat M. (1992). The utilization of seaweed meals as binding agents in pelleted feeds for snakehead (*Chalzna striatzcs*) fry and their effects on growth. Aquaculture.

[89] O’Sullivan L., Murphy B., McLoughlin P., Duggan P., Lawlor P.G., Hughes H., Gardiner G.E. (2010). Prebiotics from marine macroalgae for human and animal health applications. Mar. Drugs.

[90] Ghosh K., Ray A.K. (2018). Applications of plant ingredients for tropical and subtropical freshwater finfish: Possibilities and challenges. Rev. Aquac..

[91] Vizcaíno A.J., Fumanal M., Sáez M.I., Martínez T.F., Moriñigo M.A., Fernández-díaz C. (2019). Evaluation of *Ulva ohnoi* as functional dietary ingredient in juvenile senegalese sole (*Solea senegalensi*): Effects on the structure and functionality of the intestinal mucosa. Algal Res..

[92] Vigors S., O’Doherty J.V., Rattigan R., McDonnell M.J., Rajauria G., Sweeney T. (2020). Effect of a Laminarin Rich Macroalgal Extract on the Caecal and Colonic Microbiota in the Post-Weaned Pig. Mar. Drugs.

[93] Vizcaíno A.J., Mendes S.I., Varela J.L., Ruiz-Jarabo I., Rico R., Figueroa F.L., Abdala R., Moriñigo M.Á., Mancera J.M., Alarcón F.J. (2015). Growth, tissue metabolites and digestive functionality in *Sparus aurata* juveniles fed different levels of macroalgae, *Gracilaria cornea* and *Ulva rigida*. Aquac. Res..

[94] Ashour M., Mabrouk M.M., Ayoub H.F., El-feky M.M.M.M., Zaki S.Z. (2020). Effect of dietary seaweed extract supplementation on growth, feed utilization, hematological indices, and non-specific immunity of Nile tilapia, *Oreochromis niloticus* challenged with *Aeromonas hydrophila*. J. Appl. Phycol..

[95] Shi Q., Rong H., Hao M., Zhu D., Aweya J.J., Li S. (2019). Effects of dietary *Sargassum horneri* on growth performance, serum biochemical parameters, hepatic antioxidant status, and immune responses of juvenile black sea bream *Acanthopagrus schlegelii*. J. Appl. Phycol..

[96] Sajina K.A., Sahu N.P., Varghese T., Jain K.K. (2019). Fucoidan-rich *Sargassum wightii* extract supplemented with α -amylase improve growth and immune responses of *Labeo rohita* (Hamilton, 1822) fingerlings. J. Appl. Phycol..

[97] Sattanathan G., Palanisamy T., Padmapriya S., Anand V., Park S., Ho I., Balasubramanian B. (2020). Influences of dietary inclusion of algae *Chaetomorpha aerea* enhanced growth performance, immunity, haematological response and disease resistance of *Labeo rohita* challenged with *Aeromonas hydrophila*. Aquac. Rep..

[98] Abdel-Tawwab M., Eissa E.H., Tawfik W.A., Elnabi H.E.A., Saadony S., Bazina W.K., Ahmed R.A. (2022). Dietary curcumin nanoparticles promoted the performance, antioxidant activity, and humoral immunity, and modulated the hepatic and intestinal histology of Nile tilapia fingerlings. Fish Physiol. Biochem..

[99] de Oliveira M.N., Freitas A.L.P., Carvalho A.F.U., Sampaio T.M.T., Farias D.F., Teixeira D.I.A., Gouveia S.T., Pereira J.G., de Castro Catanho de Sena M.M. (2009). Nutritive and non-nutritive attributes of washed-up seaweeds from the coast of Ceará, Brazil. Food Chem..

[100] Sharawy Z., Ashour M., Abbas E.M., Ashry O.A. (2020). Effects of dietary marine microalgae, *Tetraselmis suecica*, on production, gene expression, protein markers and bacterial count of Pacific white shrimp *Litopenaeus vannamei*. Aquac. Res..

[101] Moutinho S., Linares F., Rodríguez J.L., Sousa V., Valente L.M.P. (2018). Inclusion of 10% seaweed meal in diets for juvenile and on-growing life stages of senegalese sole (*Solea senegalensis*). J. Appl. Phycol..

[102] Queiroz A., Pereira R., Domingues A. Effect of seaweed supplementation on growth performance, immune and oxidative stress responses in gilthead seabream (*Sparus aurata*). Proceedings of the International Meeting on Marine Research.

[103] Yu Y., Chen W., Liu Y., Niu J., Chen M., Tian L. (2016). Effect of different dietary levels of *Gracilaria lemaneiformis* dry power on growth performance, haematological parameters and intestinal structure of juvenile pacific white shrimp (*Litopenaeus vannamei*). Aquaculture.

[104] Dworjanyn S.A., Pirozzi I., Liu W. (2007). The effect of the addition of algae feeding stimulants to artificial diets for the sea urchin *Tripneustes gratilla*. Aquaculture.

[105] Tantikitti C. (2014). Feed Palatability and the alternative protein sources in shrimp feed. Songklanakarin J. Sci. Technol..

[106] Al-souti A., Gallardo W., Claereboudt M., Mahgoub O. (2019). Attractability and palatability of formulated diets incorporated with chicken feather and algal meals for juvenile gilthead seabream, *Sparus aurata*. Aquac. Rep..

[107] Bowker J. (2013). Attractant Properties of Chemical Constituents of the Green Macroalga *Ulva* and Their Response Effects on the Commercially Important Sea Urchin *Tripneustes gratilla*. Bachelor’s Thesis.

[108] Van Alstyne K.L., Wolfe G.V., Freidenburg T.L., Neill A., Hicken C. (2001). Activated defense systems in marine macroalgae: Evidence for an ecological role for DMSP cleavage. Mar. Ecol. Prog. Ser..

[109] Rajauria G., Tiwari B.K., Troy D.J. (2015). Seaweeds: A sustainable feed source for livestock and aquaculture. Seaweed Sustainability.

[110] Cruz E., Ricque D., Tapia M., Guajardo C., Obaldo L., Velasco M., Carrasco A. (2020). Water Stability, Texture of Shrimp Feeds Formulated with Natural, Synthetic Binders.

[111] Moura P.C., Fernandes J.M., Diniz M.S., Fetter V., Vassilenko V. (2023). Differentiation of the organoleptic volatile organic compound profile of three edible seaweeds. Metabolites.

[112] Marinho G., Nunes C., Sousa-Pinto I., Pereira R., Rema P., Valente L.M.P. (2013). The IMTA-cultivated chlorophyta *Ulva* spp. as a sustainable ingredient in Nile tilapia (*Oreochromis niloticus*) diets. J. Appl. Phycol..

[113] Pereira R., Valente L.M.P., Sousa-pinto I., Rema P. (2012). Apparent nutrient digestibility of seaweeds by rainbow trout (*Oncorhynchus mykiss*) and Nile tilapia (*Oreochromis niloticus*). Algal Res..

[114] Halver J.E. (2002). Nutrient flow and retention. Fish Nutrition.

[115] Montgomery W.L., Gerking S.D. (1980). Marine macroalgae as foods for fishes: An evaluation of potential food quality. Environ. Biol. Fish..

[116] Hidalgo M.C., Urea E., Sanz A. (1999). Comparative study of digestive enzymes in fish with different nutritional habits. Proteolytic and amylase activities. Aquaculture.

[117] Mwendwa R., Wawire M., Kahenya P. (2023). Effect of dietary supplementation with seaweed on growth and nutritional quality of Nile tilapia. J. Agric. Sci. Technol..

[118] Radwan M., El-sharkawy M.A., Negm M.A., Mohammadein A. (2022). Dual effect of dietary seaweed of extract nanoparticles (GNS) with bionanocomposite cellulose acetate membranes (CA/Bio-AgNps) on growth performance and health status of Nile tilapia (*Oreochromis niloticus*): Specification on feed utilization, immune system, and antiparasitic action. Front. Mar. Sci..

[119] Villa-Arce M.Á., Muñoz-Ochoa M., Hernández-Carmona G., Mendoza-Cruz M., Godínez-Pérez C.A., Vélez-Arellano N. (2023). Formulated algae-based feed with low polyphenol content and its effect on the feeding preference of juvenile blue abalone *Haliotis fulgens*. J. Appl. Phycol..

[120] Jeong J., Hwang S.J., Han M.H., Lee D., Yoo J.S., Choi I. (2017). Fucoidan inhibits lipopolysaccharide-induced inflammatory responses in RAW 264.7 macrophages and zebrafish larvae. Mol. Cell. Toxicol..

[121] El-boshy M., El-ashram A., Risha E., Abdelhamid F., Zahran E., Gab-alla A. (2014). Dietary fucoidan enhance the non-specific immune response and disease resistance in African catfish, *Clarias gariepinus* immunosuppressed by cadmium chloride. Vet. Immunol. Immunopathol..

[122] Sony N.M., Hossain S., Ishikawa M., Koshio S., Yokoyama S. (2020). Efficacy of mozuku fucoidan in alternative protein-based diet to improve growth, health performance, and stress resistance of juvenile red seabream, *Pagrus major*. Fish Physiol. Biochem..

[123] Traifalgar R.F., Kira H., Tung H.T., Raafat F., Michael, Laining A., Yokoyama S., Ishikawa M., Koshio S. (2010). Influence of dietary fucoidan supplementation on growth and immunological response of juvenile *Marsupenaeus japonicus*. J. World Aquac. Soc..

[124] Tuller J., De Santis C., Jerry D.R. (2014). Dietary influence of fucoidan supplementation on growth of *Lates calcarifer* (Bloch). Aquac. Res..

[125] Adel M., Hossein A., Dawood M.A.O., Karimi B. (2021). Dietary *Gracilaria persica* mediated the growth performance, fillet colouration, and immune response of Persian sturgeon (*Acipenser persicus*). Aquaculture.

[126] Abdelhamid A.F., Ayoub H.F., Abd El-Gawad E.A., Abdelghany M.F., AbdelTawwab M. (2021). Potential effects of dietary seaweeds mixture on the growth performance, antioxidant status, immunity response, and resistance of striped catfish (*Pangasianodon hypophthalmus*) against *Aeromonas hydrophila* infection. Fish. Shellfish Immunol..

[127] Antunes M., Neves M., Pires D., Passos R., do Carmo B., Tchobanov C.F., Forte S., Vaz M., Baptista T., Tecelão C. (2023). Proximate Composition and Fatty Acid Profile of Gilthead Seabream (*Sparus aurata*) Fed with Pelvetia canaliculate Supplemented Diets: An Insight towards the Valorization of Seaweed Biomass. Foods.

[128] Xuan X., Li W., Zhu W., Wang S. (2019). Effects of different levels of macroalga *Gracilaria lemaneiformis* on growth performance and feed utilization on the red sea bream, *Pagrosomus major*. J. Appl. Phycol..

[129] Zhongbao L.I., Huan Y., Jingbo S. (2018). Growth performance, digestive enzyme activities and serum nonspecific immunity of the red tilapia (*Oreochromis mossambicus* × *Oreochromis niloticus*) fed diets supplemented with ultrafi Ne powder of *Enteromopha prolifera*. J. Oceanol. Limnol..

[130] Younis E.M., Al-quffail A.S., Al-asgah N.A., Al-hafedh Y.S. (2017). Effect of dietary fish meal replacement by red algae, *Gracilaria arcuata*, on growth performance and body composition of Nile tilapia *Oreochromis niloticus*. Saudi J. Biol. Sci..

[131] Morshedi V., Bahabadi M.N., Sotoudeh E., Azodi M., Hafezieh M. (2018). Nutritional evaluation of *Gracilaria pulvinata* as partial substitute with fish meal in practical diets of barramundi (*Lates calcarifer*). J. Appl. Phycol..

[132] Lobo G., Pereira L.F., Gonçalves J.F.M., Peixoto M.J., Ozo´rio R.O.A. (2018). Effect of dietary seaweed supplementation on growth performance, antioxidant and immune responses in European seabass (*Dicentrarchus labrax*) subjected to rearing temperature and salinity oscillations. Int. Aquat. Res..

[133] Madibana M., Mlambo V., Lewis B., Fouché C. (2017). Effect of graded levels of dietary seaweed (*Ulva* sp.) on growth, hematological and serum biochemical parameters in dusky kob, *Argyrosomus japonicus*, Sciaenidae. Egypt. J. Aquat. Res..

[134] Shpigel M., Guttman L., Shauli L., Odintsov V., Harpaz S. (2017). *Ulva lactuca* from an integrated multi-trophic aquaculture (IMTA) biofilter system as a protein supplement in gilthead seabream (*Sparus aurata*) diet. Aquaculture.

[135] Sotoudeh E., Jafari M. (2017). Effects of dietary supplementation with red seaweed, *Gracilaria pygmae* on growth, carcass composition and hematology of juvenile rainbow trout, *Oncorhynchus mykiss*. Aquac. Int..

[136] Peixoto M.J., Salas-leitón E., Brito F., Pereira L.F., Svendsen J.C., Baptista T., Pereira R., Abreu H., Reis P.A., Fernando J. (2017). Effects of dietary *Gracilaria* sp. and *Alaria* sp. supplementation on growth performance, metabolic rates and health in meagre (*Argyrosomus regius*) subjected to pathogen infection. J. Appl. Phycol..

[137] Zhu D., Wen X., Li S., Xuan X., Li Y. (2017). Evaluation of the red alga *Gracilaria lemaneiformis* and brown alga *Sargassum horneri* as ingredients in diets for white spotted snapper *Lutjanus stellatus* akazaki juveniles. J. Appl. Phycol..

[138] Hussein E.E.M. (2017). Effect of seaweed supplemented diets on Nile tilapia, *Oreochromis niloticus* performance. Int. J. Fish. Aquat. Stud..

[139] Wan A.H.L., Soler-vila A., Keeffe D.O., Casburn P., Fitzgerald R., Johnson M.P. (2016). The inclusion of *Palmaria palmata* macroalgae in Atlantic salmon (*Salmo salar*) diets: Effects on growth, haematology, immunity and liver function. J. Appl. Phycol..

[140] Zhu D., Wen X., Xuan X. (2016). The green alga *Ulva lactuca* as a potential ingredient in diets for juvenile white spotted snapper *Lutjanus stellatus* akazaki. J. Appl. Phycol..

[141] Zeraatpisheh F., Firouzbakhsh F., Khalili K.J. (2018). Effects of the macroalga *Sargassum angustifolium* hot water extract on hematological parameters and immune responses in rainbow trout (*Oncohrynchus mykiss*) infected with *Yersinia rukeri*. J. Appl. Phycol..

[142] Peixoto M.J., Magnoni L., Gonçalves J.F.M., Twijnstra R.H., Kijjoa A., Pereira R., Palstra A.P., Ozório R.O.A. (2019). Effects of dietary supplementation of *Gracilaria* sp. extracts on fillet quality, oxidative stress, and immune responses in European seabass (*Dicentrarchus labrax*). J. Appl. Phycol..

[143] Abdelrhman A.M., Ashour M., Al-zahaby M.A., Sharawy Z.Z., Nazmi H., Zaki M.A.A., Ahmed N.H., Ahmed S.R., El-haroun E., Van Doan H. (2022). Effect of polysaccharides derived from brown macroalgae *Sargassum dentifolium* on growth performance, serum biochemical, digestive histology and enzyme activity of hybrid red tilapia. Aquac. Rep..

[144] Ferreira M., Larsen B.K., Granby K., Cunha S.C., Fernandes J.O., Nunes M.L., Marques A., Dias J., Castro L.F.C., Valente L.M.P. (2020). Diets supplemented with *Saccharina latissima* influence the expression of genes related to lipid metabolism and oxidative stress modulating rainbow trout (*Oncorhynchus mykiss*) fillet composition. Food Chem. Toxicol..

[145] Xie D., Li X., You C., Wang S., Li Y. (2019). Supplementation of macroalgae together with non-starch polysaccharide-degrading enzymes in diets enhanced growth performance, innate immune indexes, and disease resistance against *Vibrio parahaemolyticus* in rabbitfish *Siganus canaliculatus*. J. Appl. Phycol..

[146] Valente L.M.P., Araújo M., Batista S., Peixoto M.J., Sousa-pinto I., Brotas V., Cunha L.M., Rema P. (2016). Carotenoid deposition, flesh quality and immunological response of Nile tilapia fed increasing levels of IMTA-cultivated *Ulva* spp. J. Appl. Phycol..

[147] Rajendran P., Subramani P.A., Michael D. (2016). Polysaccharides from marine macroalga, *Padina gymnospora* improve the nonspecific and specific immune responses of *Cyprinus carpio* and protect it from different pathogens. Fish Shellfish Immunol..

[148] Siddik M.A.B., Rahman M.M., Anh N.T.N., Nevejan N., Bossier P. (2015). Seaweed, *Enteromorpha intestinalis*, as a diet for Nile tilapia *Oreochromis niloticus* fry. J. Appl. Aquac..

[149] Choi Y., Kim K., Han H., Nam T., Lee B. (2014). Dietary *Hizikia fusiformis* glycoprotein-induced IGF-I and IGFBP-3 associated to somatic growth, polyunsaturated fatty acid metabolism, and immunity in juvenile olive flounder *Paralichthys olivaceus*. Comp. Biochem. Physiol. Part A.

[150] Kim K., Kim S., Khosravi S., Rahimnejad S., Lee K. (2014). Evaluation of *Sargassum fusiforme* and *Ecklonia cava* as dietary additives for olive flounder (*Paralichthys olivaceus*). Turk. J. Fish. Aquat. Sci..

[151] Ragaza J.A.R., Amauag R.E.M., Oshio S.K. (2013). Comparative effects of dietary supplementation levels of *Eucheuma denticulatum* and *Sargassum fulvellum* in diet of juvenile japanese flounder *Paralichthys olivaceus*. Aquac. Sci..

[152] Kanimozhi S., Krishnaveni M., Deivasigmani B., Rajasekar T., Priyadarshni P. (2013). Immunomo-stimulation effects of *Sargassum whitti* on *Mugil cephalus* against *Pseudomonas fluorescence*. Int. J. Curr. Microbiol. App. Sci..

[153] Xu S., Zhang L., Wu Q., Liu X. (2011). Evaluation of dried seaweed *Gracilaria lemaneiformis* as an ingredient in diets for teleost fish *Siganus canaliculatus*. Aquac. Int..

[154] Kim S., Lee K. (2008). Effects of dietary kelp (*Ecklonia cava*) on growth and innate immunity in juvenile olive flounder *Paralichthys olivaceus* (Temminck et Schlege). Aquac. Res..

[155] Cheng A., Tu C., Chen Y., Nan F., Chen J. (2007). The immunostimulatory effects of sodium alginate and iota-carrageenan on orange-spotted grouper *Epinephelus coicoides* and its resistance against *Vibrio alginolyticus*. Fish Shellfish Immunol..

[156] Pham M.A., Lee K., Lee B., Lim S., Kim S., Lee Y., Heo M., Lee K. (2006). Effects of dietary *Hizikia fusiformis* on growth and immune responses in juvenile olive flounder (*Paralichthys olivaceus*). Asian-Aust. J. Anim. Sci..

[157] Wassef E.A., El-sayed A. (2005). Evaluation of *Pterocladia* (Rhodophyta) and *Ulva* (Chlorophyta) meals as additives to gilthead sea bream *Sparus aurata* diets. Egypt. J. Aquat. Res..

[158] Stentiford G.D., Sritunyalucksana K., Flegel T.W., Bryony A., Williams P., Withyachumnarnkul B., Itsathitphaisarn O., Bass D. (2017). New paradigms to help solve the global aquaculture disease crisis. PLoS Pathog..

[159] Cabello F.C. (2006). Heavy use of prophylactic antibiotics in aquaculture: A growing problem for human and animal health and for the environment. Environ. Microbiol..

[160] Baquero F., Martı´nez J.-L., Cantón R. (2008). Antibiotics and antibiotic resistance in water environments. Curr. Opin. Biotechnol..

[161] Lulijwa R., Rupia E.J., Alfaro A.C. (2020). Antibiotic use in aquaculture, policies and regulation, health and environmental risks: A review of the top 15 major producers. Rev. Aquac..

[162] Done H.Y., Venkatesan A.K., Halden R.U. (2015). Does the recent growth of aquaculture create antibiotic resistance threats different from those associated with land animal production in agriculture?. AAPS J..

[163] Mendonça A., VT R., Monserrat J., Romano L., Tesser M. (2019). The inclusion of algae *Gracilaria domingensis* in the diet of mullet juveniles (*Mugil liza*) improves the immune response. J. Appl. Aquac..

[164] Abo-Raya M.H., Alshehri K.M., Abdelhameed R.F.A., Elbialy Z.I., Elhady S.S., Mohamed R.A. (2021). Assessment of growth- related parameters and immune-biochemical profile of Nile tilapia (*Oreochromis niloticus*) fed dietary *Ulva fasciata* extract. Aquac. Res..

[165] Zhao W., Liu H.F.Z., Zhang J.C.C., Niu B.G.J. (2021). Responses in growth performance, enzymatic activity, immune function and liver health after dietary supplementation of *Porphyridium* sp. in juvenile golden pompano (*Trachinotus ovatus*). Aquac. Nutr..

[166] Rodrigues M.V., Zanuzzo F.S., De Oliveira C.A.F., Sima P., Vetvicka V. (2020). Development of fish immunity and the role of β-glucan in immune responses. Molecules.

[167] Wang C., Hu W., Wang L., Qiao H., Wu H., Xu Z. (2019). Effects of dietary supplementation with *Sargassum horneri* meal on growth performance, body composition, and immune response of juvenile turbot. J. Appl. Phycol..

[168] Maldonado-Miranda J.J., Castillo-Pérez L.J., Ponce-Hernández A., Carranza-Álvarez C. (2022). Summary of economic losses due to bacterial pathogens in aquaculture industry. Bacterial Fish Diseases.

[169] Yeganeh S., Adel M. (2019). Effects of dietary algae (*Sargassum ilicifolium*) as immunomodulator and growth promoter of juvenile great sturgeon (*Huso huso* Linnaeus, 1758). J. Appl. Phycol..

[170] Fumanal M., Di Zeo D.E., Anguís V., Fernández-diaz C., Alarcón J., Piñera R., Albaladejo-riad N., Esteban M.A., Miguel A., Balebona M.C. (2020). Inclusion of dietary *Ulva ohnoi* 5% modulates Solea senegalensis immune response during *Photobacterium damselae* subsp. piscicida infection. Fish Shellfish Immunol..

[171] Lee P., Wen C.M., Nan F.H., Yeh H.Y., Lee M.C. (2020). Immunomodulatory effects of *Sarcodia suiae* water extracts on Nile tilapia *Oreochromis niloticus* and its resistance against *Streptococcus agalactiae*. Fish Shellfish Immunol..

[172] Leonard S.G., Sweeney T., Bahar B., Lynch B.P., Doherty J.V.O. (2011). Effects of dietary seaweed extract supplementation in sows and post-weaned pigs on performance, intestinal morphology, intestinal microflora and immune status. Br. J. Nutr..

[173] Narasimhan M.K., Pavithra S.K., Krishnan V. (2013). In vitro analysis of antioxidant, antimicrobial and antiproliferative activity of *Enteromorpha antenna*, *Enteromorpha linza* and *Gracilaria corticata* extracts. Jundishapur J. Nat. Pharm. Prod..

[174] Devi G.K., Manivannan K., Thirumaran G., Rajathi A.A., Anantharaman P. (2011). In vitro antioxidant activities of selected seaweeds from southeast coast of India. Asian Pac. J. Trop. Med..

[175] Martínez-páramo S., Diogo P., Dinis M.T., Soares F., Sarasquete C., Cabrita E. (2013). Effect of two sulfur-containing amino acids, taurine and hypotaurine in European sea bass (*Dicentrarchus labrax*) sperm cryopreservation. Cryobiology.

[176] Kannan G., Saker K.E., Terrill T.H., Kouakou B., Galipalli S., Gelaye S. (2007). Effect of seaweed extract supplementation in goats exposed to simulated preslaughter stress. Small Rumin. Res..

[177] Makkar H.P., Tran G., Heuzé V., Giger-Reverdin S., Lessire M., Lebas F., Ankers P. (2015). Seaweeds for livestock diets: A review. Anim. Feed Sci. Technol..

[178] Montalban-Arques A., De Schryver P., Bossier P., Gorkiewicz G., Mulero V., Gatlin D.M., Galindo-Villegas J. (2015). Selective manipulation of the gut microbiota improves immune status in vertebrates. Front. Immunol..

[179] Gisbert E., Ortiz-Delgado J.B., Sarasquete C. (2008). Nutritional cellular biomarkers in early life stages of fish. Histol. Histopathol..

[180] Zeynali M., Bahabadi M., Morshedi V., Qasemi A., Torfi Mozanzadeh M. (2021). Effects of partial replacement of macroalgae (*Sargassum ilicifolium*) with fish meal on intestinal tissue structure in Asian seabass (*Lates calcarifer*). Vet. Res. Biol. Prod..

[181] Rombout J.H.W.M., Abelli L., Picchietti S., Scapigliati G., Kiron V. (2011). Teleost intestinal immunology. Fish Shellfish Immunol..

[182] Abdel-mawla M.S., Magouz F.I., Khalafalla M.M., Amer A.A., Soliman A.A., Zaineldin A.I., Gewaily M.S., Dawood M.A.O. (2023). Growth performance, intestinal morphology, blood biomarkers, and immune response of Thinlip grey mullet (*Liza ramada*) fed dietary laminarin supplement. J. Appl. Phycol..

[183] Passos R., Patrícia A., Ferreira I., Pires P., Pires D., Gomes E., Santos P., Simões M., Afonso C. (2021). Effect on health status and pathogen resistance of gilthead seabream (*Sparus aurata*) fed with diets supplemented with *Gracilaria gracilis*. Aquaculture.

[184] Batista S., Pereira R., Oliveira B., Baião L.F., Jessen F., Tulli F., Messina M., Silva J.L., Abreu H., Valente L.M.P. (2020). Exploring the potential of seaweed *Gracilaria gracilis* and microalga *Nannochloropsis oceanica*, single or blended, as natural dietary ingredients for European seabass *Dicentrarchus labrax*. J. Appl. Phycol..

[185] Knoop K.A., Newberry R.D. (2018). Goblet cells: Multifaceted players in immunity at mucosal surfaces. Mucosal Immunol..

[186] Niu J., Chen X., Lu X., Jiang S., Lin H., Liu Y., Huang Z., Wang J., Wang Y., Tian L. (2015). Effects of different levels of dietary wakame (*Undaria pinnatifida*) on growth, immunity and intestinal structure of juvenile *Penaeus monodon*. Aquaculture.

[187] Silva D.M., Valente L.M.P., Pereira R., Pires M.A., Seixas F., Rema P. (2015). Evaluation of IMTA-produced seaweeds (*Gracilaria*, *Porphyra*, and *Ulva*) as dietary ingredients in Nile tilapia, *Oreochromis niloticus* L., juveniles. Effects on growth performance and gut histology. J. Appl. Phycol..

[188] Abdel-warith A.A., Younis E.M.I., Al-asgah N.A. (2016). Potential use of green macroalgae *Ulva lactuca* as a feed supplement in diets on growth performance, feed utilization and body composition of the African catfish, *Clarias gariepinus*. Saudi J. Biol. Sci..

[189] Mota C.S.C., Pinto O., Sá T., Ferreira M., Delerue-matos C., Cabrita A.R.J., Almeida A., Abreu H., Silva J., Fonseca A.J.M. (2023). A commercial blend of macroalgae and microalgae promotes digestibility, growth performance, and muscle nutritional value of European seabass (*Dicentrarchus labrax* L.) juveniles. Front. Nutr..

[190] Cui H., Wang Z., Liu J., Wang Y., Wang Z., Fu J., Wan Z., Li R., Li Q., Helen J. (2020). Effects of a highly purified fucoidan from *Undaria pinnatifida* on growth performance and intestine health status of Gibel carp *Carassius auratus gibelio*. Aquac. Nutr..

[191] Nordvi M.F., Løvmo S.D., Bringslid H.I., Whatmore P., Sundh H., Reitan K.I., Aachmann F.L., Olsen R.E. (2023). Fucoidan from *Undaria pinnatifida* mitigates intestinal inflammation in Atlantic salmon (*Salmo salar*). Aquaculture.

[192] Mahgoub H.A., El-adl M.A.M., Ghanem H.M., Martyniuk C.J. (2020). The effect of fucoidan or potassium permanganate on growth performance, intestinal pathology, and antioxidant status in Nile tilapia (*Oreochromis niloticus*). Fish Physiol. Biochem..

[193] Van Vo B., Siddik M.A., Fotedar R., Chaklader R., Hanif A., Foysal J., Nguyen H.Q. (2020). Progressive replacement of fishmeal by raw and enzyme-treated alga, Spirulina platensis influences growth, intestinal micromorphology and stress response in juvenile barramundi, *Lates calcarifer*. Aquaculture.

[194] Pires D., Passos R., Carmo B., Tchobanov C.F., Forte S., Vaz M., Antunes M., Neves M., Tecel C., Baptista T. (2022). *Pelvetia canaliculata* as an aquafeed supplement for gilthead seabream *Sparus aurata*: A biorefinery approach for seaweed biomass valorisation. Sustainability.

[195] Guerreiro I., Magalhães R., Coutinho F., Couto A., Sousa S., Delerue-matos C. (2019). Evaluation of the seaweeds *Chondrus crispus* and *Ulva lactuca* as functional ingredients in gilthead seabream (*Sparus aurata*). J. Appl. Phycol..

[196] Cerezo I.M., Fumanal M., Tapia-paniagua S.T., Bautista R., Anguís V., Fernández-díaz C., Alarcón F.J., Moriñigo M.A., Balebona M.C., Xavier R. (2022). *Solea senegalensis* bacterial intestinal microbiota is affected by low dietary inclusion of *Ulva ohnoi* diet composition and preparation. Front. Microbiol..

[197] Ferreira M., Abdelha Y., Abreu H., Silva J., Valente L.M.P., Kiron V. (2022). *Gracilaria gracilis* and *Nannochloropsis oceanica*, singly or in combination, in diets alter the intestinal microbiota of European seabass (*Dicentrarchus labrax*). Front. Mar. Sci..

[198] Tapia-paniagua S.T., Fumanal M., Anguís V., Fernández-díaz C., Alarcón F.J., Moriñigo M.A. (2019). Modulation of intestinal microbiota in *Solea senegalensis* fed low dietary level of *Ulva ohnoi*. Front. Microbiol..

[199] Gonçalves A.T., Simões M., Costa C., Passos R., Baptista T. (2022). Modulatory effect of *Gracilaria gracilis* on European seabass gut microbiota community and its functionality. Sci. Rep..

[200] Abdala- R.T., García- D.J., Rosa M., Rico M., Gómez-Pinchetti J.L., Juan P., Mancera M., Figueroa F.L., Javier F., Eduardo A. (2021). Effects of a short pulse administration of *Ulva rigida* on innate immune response and intestinal microbiota in *Sparus aurata* juveniles. Aquac. Res..

[201] Yazdanpanah M., Sotoudeh E., Mansouri Taee H., Habibi H. (2021). Dietary administration of *Sargassum angustifolium* and *Gracilaria pulvinata* extracts affect antioxidant enzyme activities and *Lactobacillus* bacterial population in intestine of rainbow trout (*Oncorhynchus mykiss*) fry. Iran. J. Fish. Sci..

[202] Silva-brito F., Alexandrino D.A.M., Jia Z., Mo Y., Kijjoa A., Abreu H., Carvalho M.F., Oz R. (2021). Fish performance, intestinal bacterial community, digestive function and skin and fillet attributes during cold storage of gilthead seabream (*Sparus aurata*) fed diets supplemented with *Gracilaria* by-products. Aquaculture.

[203] Keating C., Hinchcliffe J., Davies R., Whelan S., Wan A.H.L., Fitzgerald R.D. (2021). Temporal changes in the gut microbiota in farmed Atlantic cod (*Gadus morhua*) outweigh the response to diet supplementation with macroalgae. Anim. Microbiome.

[204] Thepot V., Slinger J., Paul N.A. (2020). Influence of seaweed supplements on the intestinal bacteria in the rabbit fish *Siganus fuscescens*: Evidence for a core microbiome. Res. Sq..

[205] Gupta S., Jep L., Abdelhafiz Y.A., Siriyappagouder P., Sørensen M., Fernandes J.M., Kiron V. (2019). Macroalga-derived alginate oiligosaccharide alters intestinal bacteria of Atlantic salmon. Front. Microbiol..

[206] Xinxu Z., Huijuan W.U., Zhongzhen L.I. (2018). Effects of dietary supplementation of *Ulva pertusa* and non- starch polysaccharide enzymes on gut microbiota of *Siganus canaliculatus*. J. Oceanol. Limnol..

[207] Rico R.M., Tejedor-Junco M.T., Tapia-Paniagua S.T., Alarcón F.J., Mancera J.M., López-Figueroa F., Balebona M.C., Abdala-Díaz R.T., Moriñigo M.A. (2016). Influence of the dietary inclusion of *Gracilaria cornea* and *Ulva rigida* on the biodiversity of the intestinal microbiota of *Sparus aurata* juveniles. Aquac. Int..

[208] Diwan A.D., Harke S.N., Archana G. (2021). Aquaculture industry prospective from gut microbiome of fish and shellfish: An overview. Anim. Physiol. Anim. Nutr..

[209] Llewellyn M.S., Boutin S., Hoseinifar S.H., Derome N., Biron D.G., Romero J., De U. (2014). Teleost microbiomes: The state of the art in their characterization, manipulation and importance in aquaculture and fisheries. Front. Microbiol..

[210] Egerton S., Culloty S., Whooley J., Stanton C., Ross R.P., Ross R.P. (2018). The gut microbiota of marine fish. Front. Microbiol..

[211] Eichmiller J.J., Hamilton M.J., Staley C., Sadowsky M.J., Sorensen P.W. (2016). Environment shapes the fecal microbiome of invasive carp species. Microbiome.

[212] Gonçalves A.T., Gallardo-Escárate C. (2017). Microbiome dynamic modulation through functional diets based on pre- and probiotics (Mannan-oligosaccharides and *Saccharomyces cerevisiae*) in juvenile rainbow trout (*Oncorhynchus mykiss*). J. Appl. Microbiol..

[213] Dawood M.A.O., Koshio S. (2020). Application of fermentation strategy in aquafeed for sustainable aquaculture. Rev. Aquac..

[214] Wilczynski W., Radlinska M., Wysujack K., Czub M., Brzeziński T., Kowalczyk G., Bełdowski J., Nogueira P., Maszczyk P. (2022). Metagenomic analysis of the gastrointestinal microbiota of *Gadus morhua callarias* L. originating from a chemical munition dump site. Toxics.

[215] Chiheb I., Hassane R., José M., Seglar D., Francisco J. (2010). Screening of antibacterial activity in marine green and brown macroalgae from the coast of Morocco. Afr. J. Biotechnol..

[216] Silva M., Vieira L., Almeida A.P., Kijjoa A. (2013). The marine macroalgae of the genus *Ulva*: Chemistry, biological activities and potential applications. Oceanography.

[217] Kong Y., Teather R., Forster R. (2010). Composition, spatial distribution, and diversity of the bacterial communities in the rumen of cows fed different forages. FEMS Microbiol. Ecol..

[218] Salonen A., Lahti L., Saloja J., Holtrop G., Korpela K., Duncan S.H., Date P., Farquharson F., Johnstone A.M., Lobley G.E. (2014). Impact of diet and individual variation on intestinal microbiota composition and fermentation products in obese men. ISME J..

[219] Hehre E.J., Meeuwig J.J. (2016). A global analysis of the relationship between farmed seaweed production and herbivorous fish catch. PLoS ONE.

[220] Xu J., Liao W., Liu Y., Guo Y., Jiang S., Zhao C. (2023). An overview on the nutritional and bioactive components of green seaweeds. Food Prod. Process. Nutr..

[221] López-vivas J.M., Riosmena-rodríguez R., Alfredo A., De Llave J., Pacheco-ruíz I., Yarish C. (2015). Growth and reproductive responses of the conchocelis phase of *Pyropia hollenbergii* (Bangiales, Rhodophyta) to light and temperature. J. Appl. Phycol..

[222] Loureiro R.R., Hurtado A.Q., Critchley A.T. (2017). Impacts of AMPEP on epiphytes and diseases in kappaphycus and eucheuma cultivation. Tropical Seaweed Farming Trends, Problems and Opportunities: Developments in Applied Phycology.

[223] Mahrose K.M., Michalak I., Ranga Rao A., Ravishankar G.A. (2022). Seaweeds for animal feed, current status, challenges, and opportunities. Sustainable Global Resources of Seaweeds.

[224] Filippini M., Baldisserotto A., Menotta S., Fedrizzi G., Rubini S., Gigliotti D., Valpiani G., Buzzi R., Manfredini S., Vertuani S. (2021). Heavy metals and potential risks in edible seaweed on the market in Italy. Chemosphere.

